# A molecular phenotypic map of malignant pleural mesothelioma

**DOI:** 10.1093/gigascience/giac128

**Published:** 2023-01-27

**Authors:** Alex Di Genova, Lise Mangiante, Alexandra Sexton-Oates, Catherine Voegele, Lynnette Fernandez-Cuesta, Nicolas Alcala, Matthieu Foll

**Affiliations:** Rare Cancers Genomics Team (RCG), Genomic Epidemiology Branch (GEM), International Agency for Research on Cancer/World Health Organisation (IARC/WHO), Lyon, 69008, France; Instituto de Ciencias de la Ingeniería, Universidad de O'Higgins, Rancagua 2840390, Chile; Facultad de Ingenieria, Centro de Modelamiento Matemático UMI-CNRS 2807, Universidad de Chile, Santiago 8370285, Chile; Rare Cancers Genomics Team (RCG), Genomic Epidemiology Branch (GEM), International Agency for Research on Cancer/World Health Organisation (IARC/WHO), Lyon, 69008, France; Department of Medicine, Stanford University, Stanford, CA 94305, USA; Rare Cancers Genomics Team (RCG), Genomic Epidemiology Branch (GEM), International Agency for Research on Cancer/World Health Organisation (IARC/WHO), Lyon, 69008, France; Rare Cancers Genomics Team (RCG), Genomic Epidemiology Branch (GEM), International Agency for Research on Cancer/World Health Organisation (IARC/WHO), Lyon, 69008, France; Rare Cancers Genomics Team (RCG), Genomic Epidemiology Branch (GEM), International Agency for Research on Cancer/World Health Organisation (IARC/WHO), Lyon, 69008, France; Rare Cancers Genomics Team (RCG), Genomic Epidemiology Branch (GEM), International Agency for Research on Cancer/World Health Organisation (IARC/WHO), Lyon, 69008, France; Rare Cancers Genomics Team (RCG), Genomic Epidemiology Branch (GEM), International Agency for Research on Cancer/World Health Organisation (IARC/WHO), Lyon, 69008, France

**Keywords:** malignant pleural mesothelioma, genomics, transcriptomics, DNA methylation, quality control, cancer tasks, tumor map

## Abstract

**Background:**

Malignant pleural mesothelioma (MPM) is a rare understudied cancer associated with exposure to asbestos. So far, MPM patients have benefited marginally from the genomics medicine revolution due to the limited size or breadth of existing molecular studies. In the context of the MESOMICS project, we have performed the most comprehensive molecular characterization of MPM to date, with the underlying dataset made of the largest whole-genome sequencing series yet reported, together with transcriptome sequencing and methylation arrays for 120 MPM patients.

**Results:**

We first provide comprehensive quality controls for all samples, of both raw and processed data. Due to the difficulty in collecting specimens from such rare tumors, a part of the cohort does not include matched normal material. We provide a detailed analysis of data processing of these tumor-only samples, showing that all somatic alteration calls match very stringent criteria of precision and recall. Finally, integrating our data with previously published multiomic MPM datasets (*n* = 374 in total), we provide an extensive molecular phenotype map of MPM based on the multitask theory. The generated map can be interactively explored and interrogated on the UCSC TumorMap portal (https://tumormap.ucsc.edu/?p=RCG_MESOMICS/MPM_Archetypes ).

**Conclusions:**

This new high-quality MPM multiomics dataset, together with the state-of-art bioinformatics and interactive visualization tools we provide, will support the development of precision medicine in MPM that is particularly challenging to implement in rare cancers due to limited molecular studies.

## Context

Malignant pleural mesothelioma (MPM) is a deadly pleural cancer with currently limited therapeutic opportunities that translate into poor outcomes for patients. The latest World Health Organization classification [1] recognizes 3 different histopathologic types: epithelioid (median overall survival of 14.4 months), biphasic (9.5 months), and sarcomatoid (5.3 months). Multiomic sequencing data [2, 3] have been key in the identification of driver genes, developing and refining the characterization of molecular profiles from initial discrete clusters to a continuum [4–6], and uncovering rare genotypes such as near-haploid genomes. Such advances have revealed the rich molecular heterogeneity in MPM and have fueled the implementation of drug trials for more tailored MPM treatments. Despite their important findings, these multiomic studies have profiled only a reduced representation of the MPM genome (primarily exomes) and have mainly focused on describing simple mutational processes (i.e., copy number alterations and point mutations). Therefore, there is still a need for comprehensive multiomic datasets including whole MPM genome sequences to allow the study of complex mutational processes (e.g., whole-genome doubling, chromothripsis, extrachromosomal DNA) that have been described in other cancer types [7–9] but not in MPM. Furthermore, understanding how genomic events impact tumor phenotypes remains poorly studied in MPM. Finally, given that MPM is a rare disease, the integration of different multiomic studies is essential for reaching the statistical power needed to derive insightful biological conclusions from complex multiomic datasets.

## Data Description

Here we describe the dataset generated by the MESOMICS project that collected more than 100 MPM tumors with extensive clinical, epidemiologic, and morphologic annotations and profiled their genome, transcriptome, and epigenome. Notably, MESOMICS prioritized the sequencing of whole MPM genomes rather than exomes, resulting in the largest set of MPM genome sequences available to date. In total, we sequenced 120 MPM tumors, among which a vast majority (105) have the 3 omics data available, and the remaining 15 samples have 1 or 2 omics data types (Supplementary Table S2). This dataset has been deposited at the EMBL-EBI European Genome-Phenome Archive (EGA accession No. EGAS00001004812) and has been used to propose a new morphomolecular classification of MPM [10]. Here, we provide a comprehensive description of data quality control and links to all bioinformatic pipelines used in the project, including state-of-the-art methodology for mutational calling in tumor-only specimens. Finally, in order to maximize the reuse potential of our MESOMICS data, we integrate our cohort with the previous multiomic studies from Bueno et al. [2] and Hmeljak et al. [3] to generate the first multicohort molecular phenotypic map for MPM based on the multitask Pareto optimum theory [11]. This interactive map provides a user-friendly way to explore the molecular data and to generate new hypotheses through custom statistical tests, based on the UCSC TumorMap portal [12]. The integrated and harmonized dataset resulting from these studies is available on GitHub [13].

Primary tumor specimens were collected from surgically resected MPM. As described in Mangiante et al. [10], among them, 13 had 2 tumor specimens collected to study intratumoral heterogeneity; we report quality controls for all samples including these 13 additional samples, but only the piece with the highest tumor content as estimated by pathological review was selected for subsequent analyses, except for analyses that specifically focused on intratumor heterogeneity. The samples used in this study belong to the French MESOBANK. Our pathologist (F.G.S., see [10]) classified all tumors following the latest World Health Organization guidance, and DNA and RNA extraction methods are described in the methods section of our recent study [10].

We provide basic clinical data (age, sex, survival) as well as exposure (asbestos, smoking) and treatment data (usage and type of chemotherapy, surgery, radiotherapy, and precision treatment) (see detailed data dictionary in Supplementary Table S2). Comorbidity data were not available, but we provide where available symptoms reported at diagnosis that are informative of the state of the patient at diagnosis (pain, pleural effusion, dyspnea, pneumothorax, coughing). Note that because of the retrospective nature of the samples from the French MESOBANK, patients were diagnosed (year of diagnosis [1998–2017], median of 2011) and treated (year of death or end of follow-up [2000–2020], median of 2013) before the results of recent promising clinical trials (MAPS [14] and Checkmate 743 [15]) and before the authorization of nivolumab and ipilimumab by the European Medicines Agency in 2022 (note that despite the MAPS trial, bevacizumab is not a standard first-line treatment in France); future studies will thus probably include more patients who underwent precision treatments and hopefully report longer survival [15].

## Quality control of omic data

### Whole-genome sequencing

Whole-genome sequencing (WGS) was performed by the Centre National de Recherche en Génomique Humaine (CNRGH, Institut de Biologie François Jacob, CEA, Evry, France) on 130 fresh-frozen MPMs, plus 54 matched-normal tissue or blood samples (matched nonneoplastic tissue was not available for the other specimens). The Illumina (San Diego, CA, USA) TruSeq DNA PCR-Free Library Preparation Kit was used for library preparation and the HiSeqX5 platform from Illumina for the sequencing as described in [10]. The raw WGS reads were scanned by the FastQC software (v.0.11.5; RRID:SCR_014583; using our nextflow [16] pipeline IARCbioinfo/fastqc-nf [17]) to determine the reads base quality, adapter content, and duplication levels. The software MultiQC (v0.9; RRID:SCR_014982) was then used to aggregate all the FastQC reports across samples.

**Figure 1:**
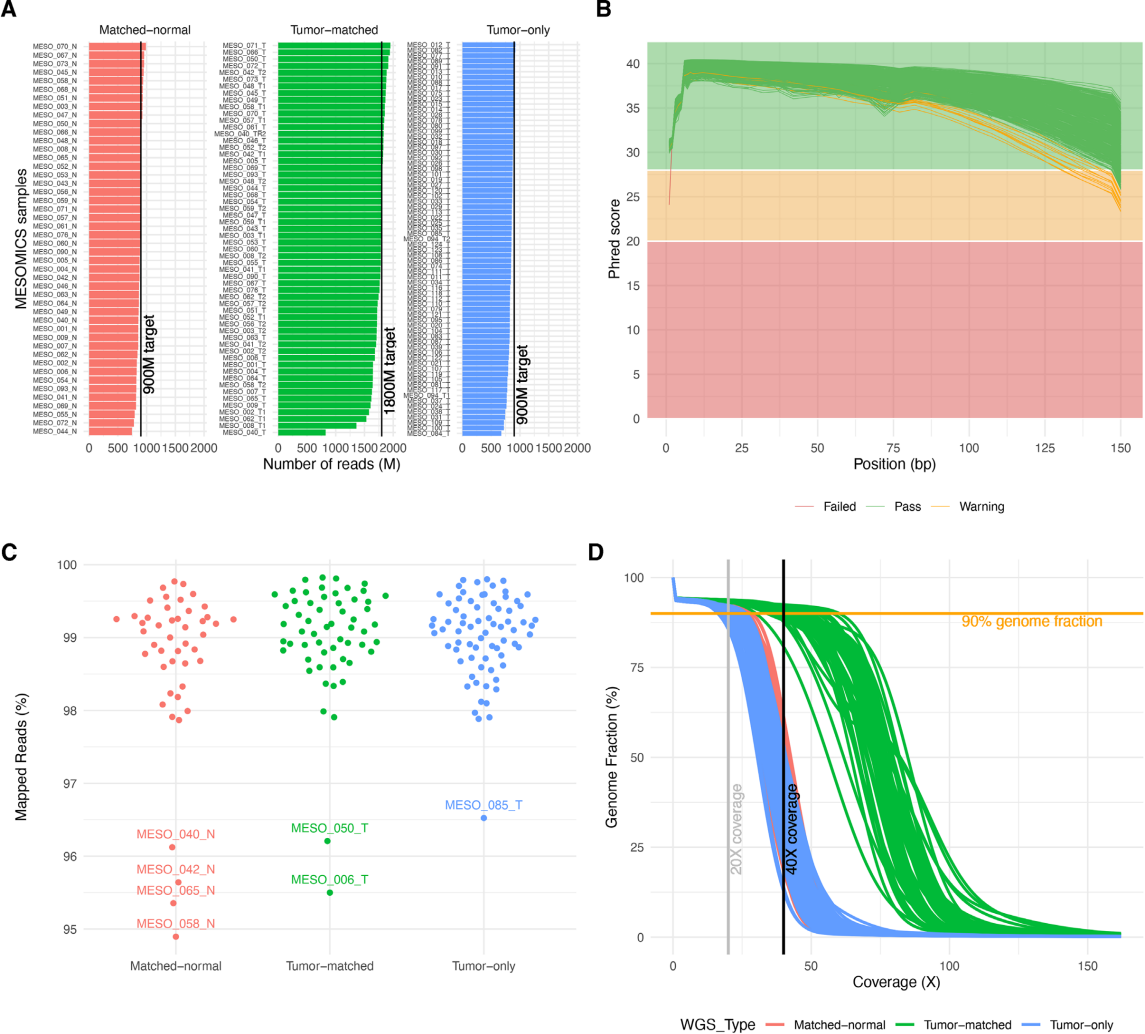
Quality control of whole-genome sequencing (WGS) data. (A) Number of reads per WGS type. (B) Mean sequence quality score as a function of the position in the read in base pairs. Green lines correspond to files that passed the most stringent quality control filters of software FastQC; orange lines correspond to files that passed a less stringent filter and red to files that did not pass the filters. (C) Percentage of aligned reads to the reference human genome. (D) Cumulative genome fraction computed directly from the BAM files.

The target read output for matched-normal tissue or blood (hereinafter called “matched-normal”) and for tumor tissues without matched-normal sample (hereinafter called “tumor-only”) was 900 million reads (∼30× genome coverage, Fig. 1A). In total, 1,800 million (∼60× genome coverage, Fig. 1A) were expected for tumor tissues with matched-normal samples (2 sequencing lanes, hereinafter called “tumor-matched”). Overall, the median number of reads obtained approached or exceeded the target read output, with median and standard deviation by sample type equal to the following: matched-normal, 889 ± 50; matched-tumor, 1,786 ± 163; and tumor-only, 853 ± 51 million reads (Fig. 1A).

All samples displayed the expected mean quality score (30Q >85% of bases) across all base positions of the read (Fig. 1B). One exception is the MESO_050_N (a matched-normal sample) that on average had a good sequence quality score (Fig. 1B) but displayed a low mean quality score for the first nucleotide of the read (24.08 Phred), which FastQC reported as a warning in the mean quality score module (Fig. 1B). In fact, the FastQC report for this sample indicated that 25.17% of bases were not called at the first nucleotide of the read, suggesting that the base-calling process struggled in interpreting the DNA bases at this position and put an N instead. However, the reverse pair-end file of this sample had the expected sequence quality score over all the read positions (Fig. 1B), and we decided then to include this sample in the subsequent analyses. The adapter content was lower than 1% for all sequenced samples (maximum 0.87% of total reads). The relative level of duplication found for every sequence per sample was on average 10.3% (min: 0% and max: 18.2%); this low level of duplication indicates that the prepared genomic libraries were diverse and likely covered a high proportion of the human genome.

Paired-end read mapping was performed with our nextflow pipeline IARCbioinfo/alignment-nf v1.0 [18]. This pipeline includes the software qualimap (v2.2.2b; RRID:SCR_001209) and MultiQC to generate comprehensive quality control (QC) statistics reports from the WGS alignment files. The mean percentage of aligned reads was 98.93% ± 0.81% (Fig. 1C). The matched-normal and tumor-only samples displayed a mean genome coverage higher than 30× (Fig. 1D). The matched-tumor displayed a mean genome coverage of 60× (Fig. 1D). Finally, 90% of the reference genome was covered by at least 22, 20, and 43 reads for matched-normal, tumor-only, and matched-tumor samples, respectively (Fig. 1D).

### RNA sequencing data

RNA sequencing (RNA-seq) was performed on 126 fresh-frozen MPM in the Cologne Center for Genomics. Libraries were prepared using the Illumina TruSeq RNA sample preparation Kit, the Illumina TruSeq PE Cluster Kit v3, and an Illumina TruSeq SBS Kit v3-HS; subsequent sequencing was carried out in an Illumina HiSeq 2000 sequencer, as described in [10].

The resulting raw reads files were processed using our nextflow RNA-seq processing pipeline IARCbioinfo/RNAseq-nf v2.3 [19], as described previously [10, 20], that performs reads trimming (Trim Galore v0.6.5; RRID:SCR_011847), and mapping to reference genome GRCh38 (gencode version 33) with STAR (v2.7.3a; RRID:SCR_004463) [21]. We also improve the alignments as described previously by performing assembly-based realignment (nextflow pipeline IARCbioinfo/abra-nf v3.0 [22]), using software ABRA2 [23] (RRID:SCR_003277) and base quality score recalibration (nextflow pipeline IARCbioinfo/BQSR-nf v1.1 [24]), using GATK v4.1.7.0 [25] (RRID:SCR_001876). Gene-level quantification was performed using software StringTie (v2.1.2; RRID:SCR_016323) (nextflow pipeline IARCbioinfo/RNAseq-transcript-nf v2.2 [26]). Quality control of the samples was performed using FastQC (v0.11.9; RRID:SCR_014583) to determine the quality of the raw reads, followed by RSeQC (v3.0.1; RRID:SCR_005275) [27] that was used to determine the alignment quality and distribution of reads over the reference genome (number of mapped reads, proportion of uniquely mapped reads). Finally, the software MultiQC (v0.9; RRID:SCR_014982) [28] was used to aggregate all QC results across samples.

**Figure 2:**
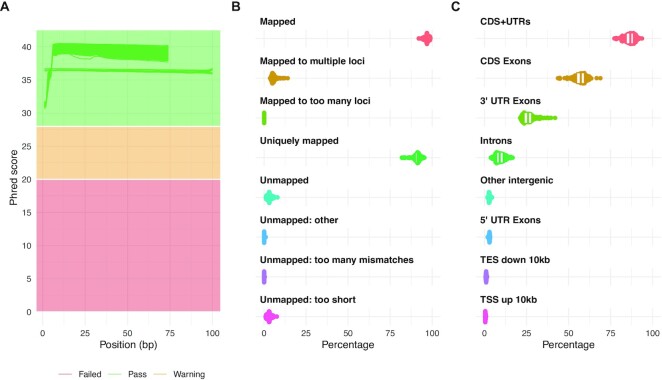
Quality control of RNA-seq data. (A) Distribution of sequence quality scores in Phred scale for 2 × 75-bp and 2 × 100-bp read pairs. (B) STAR alignment scores. (C) Distribution of reads mapped to different genomic regions.

A total of 126 samples were sequenced using 2 × 75-bp or 2 × 100-bp paired-end reads (Fig. 2A). On average, a total of 64 ± 7.4 paired-end million reads were generated with a per sequence mean quality score higher than 35 (Fig. 2A). Given the high coverage and the lower length expected for a human transcriptome, the percentage of duplicated reads was high, reaching 69% ± 5.5%, but the proportion of overrepresented sequences was low (<2%), indicating that all RNA sequenced libraries were diverse. The report of STAR alignments showed that on average, 96.8% ± 1.2% of the reads mapped to the reference genome with 91% ± 2.3% mapping to unique loci (Fig. 2B). The 3.15% ± 1.26% of unmapped reads correspond mainly to reads with a short alignment length (3.05% ± 1.24%) that might result from the trimming process (trim of adaptor or low-quality bases, Fig. 2B). Finally, as expected, most of the MESOMICS reads mapped to messenger RNA structures, including CDS and UTR regions (86.2% ± 3.1%, Fig. 2C).

### DNA methylation data

DNA methylation analyses were performed in-house for 135 MPM samples from 122 patients, and an additional 2 technical replicates and 3 adjacent normal tissues, with Infinium EPIC DNA methylation beadchip platform (Illumina), which interrogates over 850,000 CpG sites, as described in [10]. Resulting raw IDAT files were processed using our in-house workflow [29] (commit SHA bcfe876) in the R statistical programing environment using R packages minfi (v1.34.0; RRID:SCR_012830) and ENmix (v1.25.1), and consisted of the following 4 steps: preprocessing quality control, functional normalization, probe filtering, and finally β- and M-value computation.

**Figure 3:**
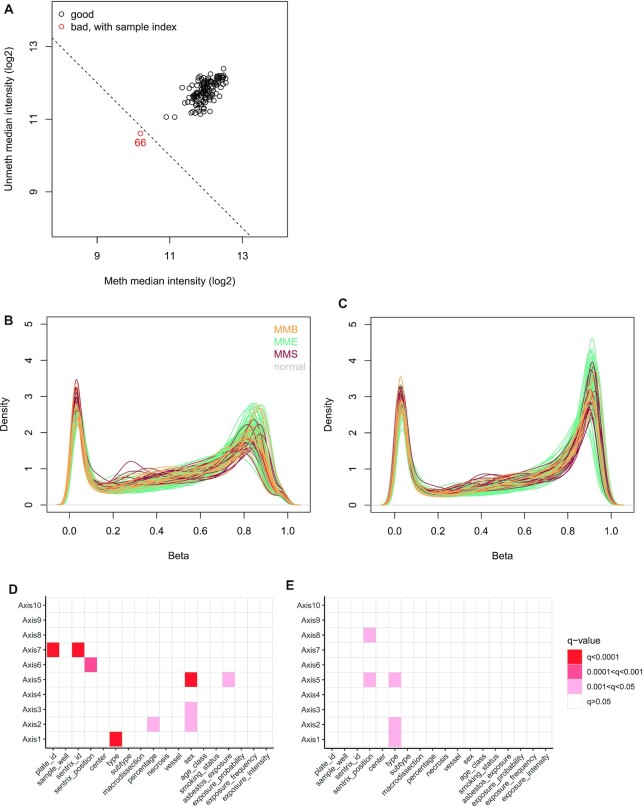
Quality control of EPIC array sequencing data. (A) Signal intensity plot. Log_2_ methylated and unmethylated median signal intensity plot of 140 samples. One sample (colored red) fell below the cutoff of 10.5 and was subsequently removed from analysis. (B) Prenormalization β density plot. The β density plot of 140 samples across 865,859 probes, colored by tumor/normal type, prior to functional normalization. (C) Postnormalization and filtering β density plot. The β density plot of 139 samples across 781,245 probes, colored by tumor/normal type, following functional normalization and removal of cross-reactive probes, sex chromosome probes, single-nucleotide polymorphism probes, and failed (*P*-detection > 0.01) probes. (D) Association of technical and clinical variables with prenormalization principal components. Association of technical and clinical variables with principal components 1 to 10, for 122 samples. Principal components calculated from M-values of 863,381 prenormalized probes. (E) Association of technical and clinical variables with postnormalization principal components. Principal components calculated from M-values of 781,245 probes following functional normalization and probe removal.

During quality control checks on the raw data, one poor-quality sample was identified when comparing per sample log_2_ methylated and unmethylated chip-wise median signal intensity (function getQC, minfi, Fig. 3A), which was subsequently removed, and all samples displayed an overall *P*-detection value < 0.01 (function detectionP, minfi). Functional normalization, probe filtering, and β- and M-value computation were performed as described in [10]. The resulting dataset consisted of β- and M-values for 139 samples across 781,245 probes, with the M-value table containing 9 -∞ values, which were replaced by the next-lowest M-value for statistical analysis. The effect of normalization and probe removal on DNA methylation profile is shown as β density plots (prenormalization in Fig. 3B and postnormalization and probe removal in Fig. 3C). Principal components analysis (PCA) was performed to detect batch effects and to examine the effect of normalization; this was performed on a reduced number of samples (*n* = 122, 1 tumor per patient, excluding technical replicates and normal tissues). Two datasets were used: (i) prenormalized, unfiltered M-values (obtained from the GenomicRatioSet, function getM, minfi) and (ii) normalized and filtered M-values. Dataset (i) contained 2,478 CpGs with at least 1 NA (Not Available) value, which were omitted before PCA, and 21,969 -∞ values were replaced with the next lowest M-value in the dataset, leaving an M-value matrix of 863,381 probes.

R package ade4 (v1.7–15) was used to calculate the first 10 principal components (function dudi.pca) across each dataset individually. We checked the association of the first 10 principal compenents (PCs) with technical (chip, position on the chip, batch, sample well, sample provider, macrodissection), clinical (sex, age class, and smoking status), morphologic (histopathologic type, subtype, tumor percentage, necrosis, and vessel level), and epidemiologic variables (asbestos exposure, exposure probability, exposure frequency, and exposure intensity) using PC regression analysis, fitting separate linear models to each principal component with each of the 18 covariables of interest and adjusted the *P*values for multiple testing (Fig. 3D and E). The first 10 principal components in the normalized, filtered methylation data were significantly associated with type (PCs 1, 2, and 5) and sentrix chip position (PC 5, PC 8). The contribution of variance in the data from technical features before normalization was more pronounced, with sentrix chip and plate also being significant (PC 7), indicating functional normalization reduced technical batch effects on DNA methylation profile while retaining biological effects such as histologic type. Before normalization, sex was significantly associated with PCs 2, 3, and 5 but not associated with any PCs in the normalized, filtered dataset. As probes on the sex chromosomes were removed after normalization, it was expected that this would reduce the effect of sex on variance in the dataset.

## WGS Variant Calling in Tumor-Only Samples

### Copy number variants

**Figure 4:**
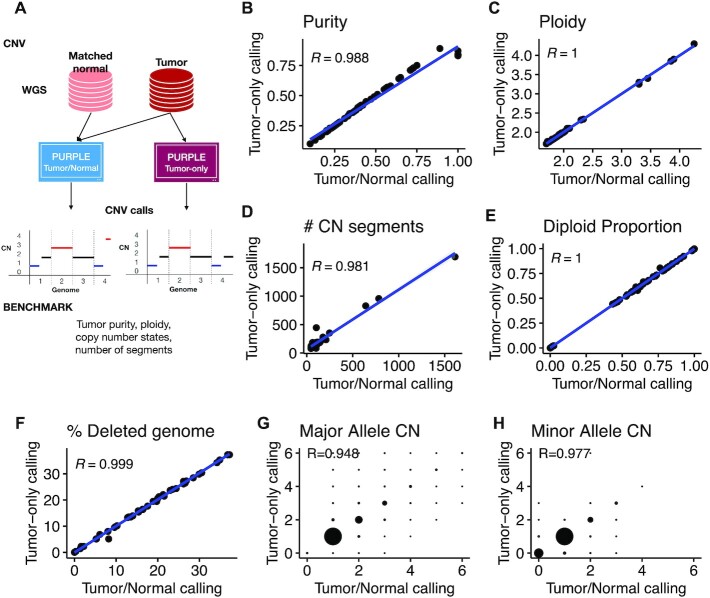
Performance of somatic copy number variant calling from tumor-only samples. (A) Schematic of the benchmarking procedure. Comparison of tumor/normal and tumor-only calling for (B) purity, (C) ploidy, (D) number of copy number segments, (E) diploid proportion, (F) percentage of deleted genome, (G) major allele copy number, and (H) minor allele copy number.

Somatic copy number alterations were called using our nextflow workflow IARCbioinfo/purple-nf v1.0 [30] that implements the PURPLE [31, 32] (RRID:SCR_022999) software for matched and tumor-only WGS samples. To assess the quality of tumor-only PURPLE calls, a total of 57 matched pairs were used as an evaluation set. Briefly, we ran PURPLE twice for each matched sample (Fig. 4A): first using as input the matched pairs and second using only the tumor WGS as input. Subsequently, we performed a direct comparison of the PURPLE tumor-only calls with their corresponding matched-pair calls for the following features: tumor purity; ploidy; number of segments; percentage of diploid, amplified, and deleted genome regions; and major and minor copy number states at the gene level (Fig. 4).

This benchmarking revealed a high concordance across all the evaluated metrics between tumor-only and matched PURPLE calls. Indeed, the agreement for purity (Fig. 4B, *R* = 0.988), ploidy (Fig. 4C, *R* = 1), number of copy number segments per tumor (Fig. 4D, *R* = 0.981), and percentage of genome changed (diploid, amplified, and deleted) exceeded a 0.98 correlation (Fig. 4E and F). Moreover, a high concordance was also observed at the gene level with major and minor copy number alleles reaching *R* > 0.94 (Fig. 4G and H). Finally, the only detected issue of tumor-only calls was observed near telomeric and centromeric regions, where artifactual focal peaks were detected (Supplementary Figure S1). These problematic regions were manually curated, and the copy number segments overlapping such regions were removed from the tumor-only calls (see list of excluded segments in Supplementary Table S1). In addition, because PURPLE does not round copy number values to 0 but rather penalizes negative values in the model fit, for all samples (both matched and tumor-only), following similar discussions with the PURPLE developers on the handling of negative values [33], we rounded slightly negative copy number estimates (in]−0.5,0[) to 0 and excluded largely negative copy number estimates (<−0.5) from subsequent analyses, because they suggest high noise in the read depth and are thus unreliable calls. Note that in total (including segments with largely negative values), we excluded only 0.26% of the total segment length.

### Calling somatic point mutations and structural variants

**Figure 5:**
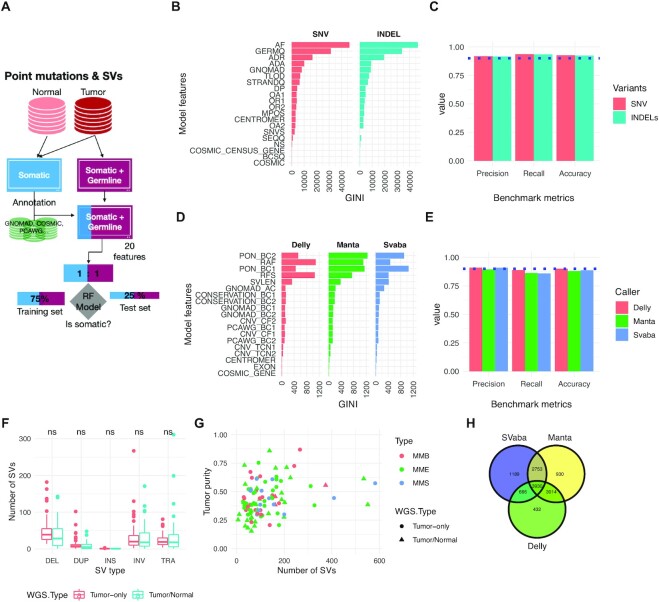
Performance of somatic point mutation and structural variant calling from tumor-only samples. (A) Schematic of the benchmarking procedure. (B) Random forest (RF) model features and their ranking for predicting somatic single-nucleotide variant (SNV) and indels. (C) Performance metrics (precision, recall, accuracy) for classifying somatic point mutations with the best-performing RF models. (D) Structural variant (SV) random forest model features and their ranking for predicting somatic SVs. (E) Performance metrics for classifying somatic SVs. (F) Number of SVs as function of WGS type. Mean comparison between WGS types was performed using a *t*-test with no significant (ns) result found. (G) Number of SVs as a function of tumor purity. A linear model (number_sv ∼Purity*WGS_type*SubType) was built to predict the number of SVs, and no significant coefficients (*P* < 0.05) were found. (H) Venn diagram of the final consensus MESOMIC SV set.

Unlike copy number variants, whereby the software (PURPLE) directly generated highly accurate results in tumor-only mode without any postprocessing, for point mutations and structural variants (SVs), direct outputs from calling pipelines and typical filters (i.e., removing variants matching germline databases) did not remove at high accuracy the germline variants present in tumor-only MPM WGS. Therefore, we trained and evaluated the performance of a supervised machine learning model based on a random forest (RF [34]) for distinguishing germline from somatic variants in tumor-only WGS (Fig. 5A, Supplementary Note 1).

Point mutations were called using Mutect2 (RRID:SCR_000559) using our nextflow pipeline IARCbioinfo/mutect-nf v2.2b [35]. The matched samples were used as input for training and evaluating the performance of the RF model (Fig. 5A) for classifying germline and somatic mutations. The RF model for point mutations includes a total of 20 features divided into 3 main classes: associated with external databases (gnomAD r3.0[36] RRID:SCR_014964 and COSMIC v90 [36] RRID:SCR_002260), genomic location/impact/signatures [37], and features obtained directly from the point mutation variant caller—Mutect2 (Fig. 5B, Supplementary Note 1). The matching of variants against reference databases was performed using bcftools (v1.10.2, annotate function RRID:SCR_005227) [38]. For training the RF model, a total of 46 tumors with matched normal MPM whole-genome sequences called with both the tumor-only and matched modes of Mutect2 [23] (RRID:SCR_000559) were used (Fig. 5A). The matched somatic calls (ground truth) were used to annotate the variants of the tumor-only WGS into germline and somatic classes.

The training and evaluation of models were performed using 75% and the remaining 25% of the dataset, respectively. A grid search revealed that the optimum parameters were mtry = 8, ntree = 1000, and nodesize = 5, reaching a model accuracy of 0.9276 in the testing set. A random forest model for single-nucleotide variants (SNVs) (rfvs01) was trained with the optimum parameters using a total of 326,388 (80%) variants (1:1 ratio). Analysis of the feature importance revealed that the allele frequency is the most discriminative feature included in the model (Fig. 5B). For indels, a random forest model (rfvi01) was built with the same optimal parameters using a total of 337,442 variants (1:1 ratio, including 305,988 SNVs and 31,454 indels) and removing the SNV feature. The performance of the optimal RF models for SNVs and indels reached an accuracy of 0.926 and 0.924, respectively (Fig. 5C). The trained RF models (rfvs01 and rfvi01) were used to classify a total of 1,454,942 variants (SNVs = 1,317,200 and indels = 137,742), of which 217,436 variants (including SNVs and indels) were classified as somatic.

Large genomic rearrangements were detected using a consensus variant calling approach including SvABA (v1.1.0) [39] (RRID:SCR_022998), Manta (v1.6.0) [40] (RRID:SCR_022997), and Delly (v0.8.3, RRID:SCR_004603) [41], followed by subsequent integration with SURVIVOR (v1.0.7) [42] (RRID:SCR_022995). Our nextflow pipeline implementing the consensus variant calling approach for matched WGS is available in our IARCbioinfo/sv_somatic_cns GitHub repository [43].

Like for point mutations, we implemented custom random forest models to distinguish at high accuracy somatic from germline SVs in tumor-only MPM samples (Fig. 5A, Supplementary Note 1). The RF models were composed of a total of 19 features based on external databases (gnomAD [36] RRID:SCR_014964 and PCAWG [44]); a custom panel of normal (PON), genomic regions (Cosmic v90 RRID:SCR_002260, Gencode v33 RRID:SCR_014966, and PhastCons [45] RRID:SCR_003204); and SV features obtained directly from each SV caller (Fig. 5D). The training (75%) and evaluation (25%) of the random forest model for each SV caller were performed using a total of 12,454, 16,720, and 12,264 SVs at 1:1 somatic: germline proportions for Delly [39] (RRID:SCR_004603), Manta [40], and SvABA [41], respectively. All 3 SV random forest models were trained using the default random forest parameters (mtry = 4, ntree = 400, and nodesize = 1). The precision, recall, and accuracy achieved by each model were 0.905 ± 0.009, 0.87 ± 0.016, and 0.889 ± 0.010, respectively (Fig. 5E). The most important features of the models were the number of PON SVs around both breakpoints, SV alternative allele frequency, SV read depth, and SV length (Fig. 5D). We performed additional comparisons by SV type, SV length, and number of SVs as a function of the purity of samples, WGS type (matched tumor-normal, tumor-only), and MPM subtype and did not observe any significant difference between SVs called in the tumor-only or matched WGS MESOMICS series (Fig. 5F and G). The SV calls for the MESOMICS tumor-only samples include a total of *n* = 8,229 SVs, which combined with the SVs called in the matched series gave a total of *n* = 12,914 (Fig. 5H).

Our results demonstrate that our methodology is highly accurate and robust to call point mutations and structural variants in tumor-only WGS datasets for which a series of matched tumor-normal samples are available. The source code and the random forest models implemented for MPM are available in our GitHub repositories IARCbioinfo/RF-mut-f [46] and IARCbioinfo/ssvht [47] for point mutations and structural variants, respectively.

## Data validation

### Multiomic sample matching

**Figure 6:**
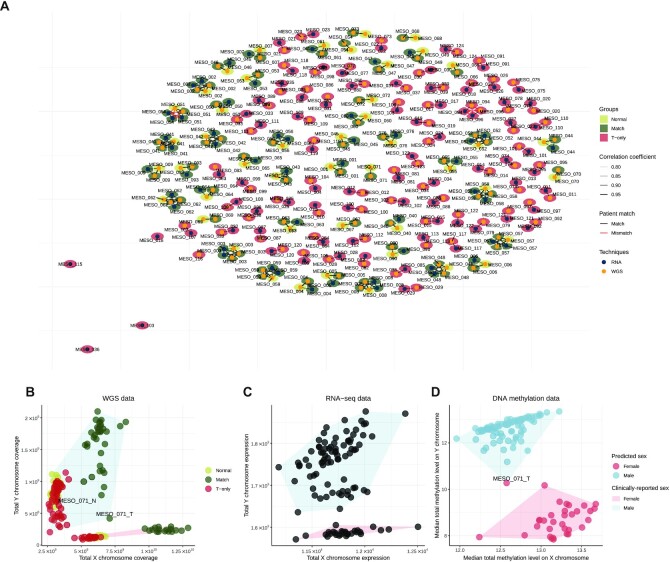
Applications of data validation using multiomics data. (A) Network of matching WGS and RNA-seq samples, as computed by software NGSCheckmate. Edge transparency corresponds to the Pearson correlation *r* between single-nucleotide polymorphism panel allelic fractions; node color and surrounding color correspond respectively to the techniques (WGS or RNA-seq) and to the tissue type (normal, matched samples, or T-only samples). (B–D) Sex reclassification and multiomic validation of reported clinical sex. (B) Total exome reads coverage on the X and Y chromosomes for each sample. (C) Total expression level of each sample on the X and Y chromosomes (in variance-stabilized read counts). (D) Median methylation array total intensity on the X and Y chromosomes. In panel (B), point colors correspond to the WGS groups: normal samples in light green, tumor samples with matched normals (Match) in dark green, and tumor samples without matched normal (T-only) in red. In each panel, filled polygons correspond to the sexes given by the clinical annotations (blue for male, red for female). In panel (D), point colors correspond to the sexes predicted by the DNA methylation QC. Samples with discordant reported clinical sex and molecular patterns on sex chromosomes are indicated.

The software NGSCheckMate [48] (RRID:SCR_022994) was used to check the match between sequencing modalities of a given MESOMICS patient. NGSCheckMate was run using our nextflow implementation IARCbioinfo/NGSCheckMate-nf v1.1a [49]. NGSCheckMate, using WGS and RNA-seq, confirmed that the majority of MESOMICS samples were correctly paired (Fig. 6A, black segments). However, NGSCheckMate discovered that the WGS of MESO_094_T and MESO_096_T matched (Fig. 6A, red segments). Further examination of these samples confirmed that both WGS had come from the same patient but were annotated differently during sample collection. In addition, the RNA-seq replicate named MESO_054_TR1 matched with the group of samples coming from patient MESO_051. After sequencing a second RNA-seq aliquot from MESO_054_T, named MESO_054_TR2, and reperforming the NGSCheckMate analysis, we confirmed a misannotation of these RNA-seq samples and proceeded to rename them as MESO_051_TR1 and MESO_051_TR2, respectively. After the aforementioned corrections, all the sequencing modalities at the sample and patient level were correctly paired for the complete MESOMICS cohort.

### Sex validation

We registered the sex (M for male or F for female) data for all the 124 patients of the MESOMICS cohort. We validated the sex annotation based on the concordance of whole-genome, transcriptome, and methylome data (Fig. 6B–D). First, the concordance between sex reported in the clinical data and WGS data was assessed by computing the total coverage on X and Y chromosomes (Fig. 6B). Interestingly, some tumors from male individuals displayed an intermediate coverage on chromosome Y between other male and female cases, compatible with the large copy losses identified in our study (e.g., the tumor from MESO_071). Second, the concordance between sex reported in the clinical data and sex chromosome gene expression patterns (transcriptome) was performed by comparing the sum of variance-stabilized read counts (vst function from R package DESeq2, v.1.14.1 RRID:SCR_015687) of each sample on the X and Y chromosomes (Fig. 6C). Third, the concordance between the sex reported in the clinical data and the methylation data was assessed using a predictor based on the median total intensity on sex chromosomes, with a cutoff of −2 log_2_ estimated copy number (function getSex from minfi, v.1.34.0 RRID:SCR_012830, Fig. 6D). The only sex discordance was observed in MESO_071 tumor sample due to somatic copy number losses in the Y chromosome, but the whole-genome sequencing from matched blood confirmed that this patient was male (Fig. 6B). In summary, the sex data of the MESOMICS cohort were validated using a multiomic approach that confirmed the sex of all the MESOMICS samples.

### Purity

Tumor purity has been estimated from 3 independent data sources: from genomic data using PURPLE, from transcriptomic data using quanTIseq [50] (RRID:SCR_022993), and through pathologic review. We performed pairwise comparisons between these 3 estimates and found significant correlations between pathologic and each molecular estimate (*q* = 8.40 × 10^−3^ and 2.49 × 10^−4^ with transcriptomic and genomic purity, respectively). The transcriptomic and genomic estimates are significantly correlated as well (*q* = 4.05 × 10^−4^, Supplementary Figure S2). Of note, 4 samples (MESO_050_T, MESO_058_T2, MESO_059_T1, and MESO_076_T) have been excluded from the analyses of genomic estimates of purity because no somatic copy number variants were identified and thus purity could not be estimated by PURPLE. The 4 samples all had low pathologic estimates ([0.1–0.4]) and moderate transcriptomic estimates of purity ([0.53–0.69]).

## An Integrative and Interactive MPM Phenotypic Map

### Task specialization analysis using Pareto

In order to integrate the MESOMICS multiomic data and investigate the association between the detected genomic events in this new large genomic cohort and the observed MPM phenotypes, we first performed a multiomic summary of MPM using MOFA [51] (RRID:SCR_022992) and, second, performed a task specialization analysis to identify MPMs with natural selection for specific cancer tasks (see [10]). We performed task specialization analyses using the well-established Pareto optimum theory (ParetoTI method) [11]. The Pareto front model has been fitted to different sets of samples using the ParetoTI R package v0.1.13 [52] (RRID:SCR_022991) on MOFA latent factors (LFs), restricted to LF1, LF2, LF3, and LF4 due to their association with survival and extreme phenotypes (see [10]). In brief, according to the theory, a molecular map would take a particular shape (polyhedra) if a trade-off exists between several cancer tasks performed by the tumors. Using MOFA axes, we found a triangle (polyhedra with 3 vertices) corresponding to *k* = 3 archetypes in the LF2–LF3 space. According to the Pareto optimum theory, this pattern results from natural selection for cancer tasks, with specialized tumors close to the vertices of the triangle (representing archetypes) and generalists in the center. We have also replicated the same analyses (MOFA and ParetoTI) on the previously published multiomic studies from Bueno et al. [2] (*n =181*fresh-frozen surgically resected primary tissue) and Hmeljak et al. [3] (*n =*73 fresh-frozen surgically resected or biopsy tissues). R scripts to prepare matrices for each omic layer, as well as scripts to run MOFA and the Pareto analysis for the 3 cohorts, are available in the GitHub repository dedicated for this data note paper [13].

### Biological interpretation of the MPM phenotypic map

We inferred each archetype's phenotype by performing integrative gene set enrichment analysis on the expression data and identified the following cancer tasks and tumor phenotypes: cell division, tumor–immune interaction, and acinar phenotype (see [10]). Tumors specialized in the cell division task displayed upregulation of pathways within the “cell division” task as reported by Hausser et al. [53] in multiple tumor types. This phenotype was enriched for nonepithelioid tumors and presented higher levels of necrosis, higher grade, high expression of hypoxia response pathways, and greater percentage of infiltrating neutrophils that are innate immune response cells. Cell division specialization was supported by the high expression levels of the proliferation marker *MKI67* and increased genomic instability. Tumors specialized in the tumor–immune interaction task carried upregulated immune-related pathways, high expression of immune checkpoint genes, and high immune infiltration with an enrichment for adaptive-response cells: lymphocytes B, T-CD8^+^, and T-reg. The last extreme phenotype was characterized by samples with acinar morphology, presenting a very structured tissue organization with epithelial cells tightly linked into tubular structures, and correlated with the presence of monocytes and natural killer cells (innate immune response cells). This phenotype presented the lowest epithelial-mesenchymal transition score [54], with overexpression of epithelial markers such as cell-adhesion molecules, corroborating the importance of tissue organization in this phenotype, and also low levels of *MKI67* expression, indicating slow growth. Altogether, these data provide a biological understanding for the molecular and phenotypic heterogeneity characteristic of MPM tumors.

### Reuse potential

The MESOMICS project represents the most comprehensive molecular characterization of MPM to date, made possible by inclusion of the largest WGS dataset yet reported, and by the depth of the analyses undertaken. Multiomics integration and biological interpretation through the lens of Pareto theory has allowed us to uncover 3 specialized MPM tumor profiles [10]. In order to replicate these findings while minimizing batch effects associated with bioinformatics data processing, we have accessed and reprocessed the raw data from previously published MPM multiomics studies [2, 3] using the same analytical procedures. A by-product of this laborious work is the creation of the largest (*n =374*samples in total) existing harmonized dataset of MPM multiomics data.

In order to maximize the reuse potential of this dataset, we have also harmonized the available clinical, epidemiologic, and morphologic data from these 3 cohorts. In addition to providing the raw data, the full list of genomic variants, and the entire matrices of expression and methylation levels, we provide a curated and harmonized list of molecular features (e.g., immune cell composition, measures of genomic instability, presence of whole-genome duplication, copy number in recurrently altered regions, driver gene mutational status, expression level of some relevant genes) across all samples (Supplementary Table S2).

**Figure 7:**
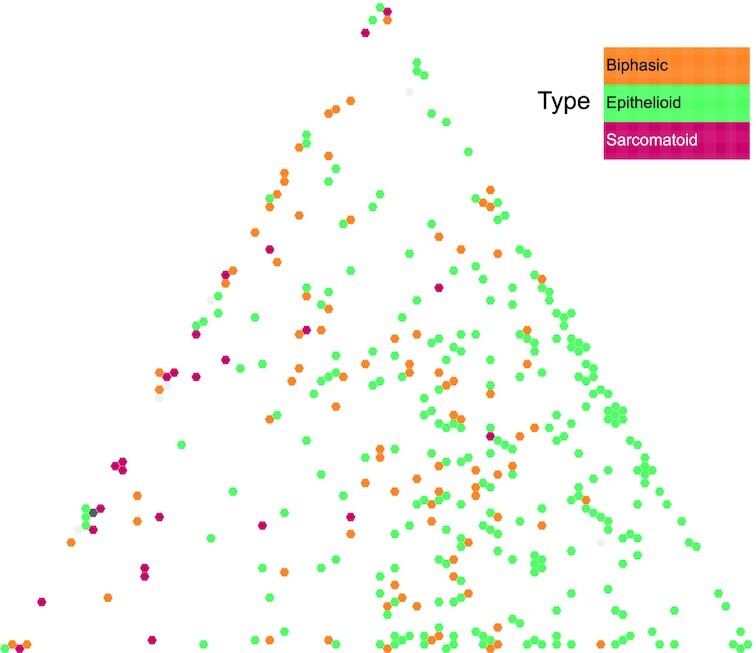
MPM molecular phenotypic map. Screen capture from the TumorMap portal, using the hexagonal grid view, each point representing a MPM sample in the triangular phenotypic space: cell division (left vertice), tumor–immune interaction (top vertice), and acinar phenotype (right vertice). Point colors correspond to the histologic types and can be interactively changed by the users on the web portal.

This MPM phenotypic map has been shared on the TumorMap web portal [12], offering an interactive visualization of these data in the tumor phenotypes space (cell division, tumor–immune interaction, and acinar phenotype), including all the harmonized clinical, morphologic, epidemiologic, and molecular data attributes mentioned above. The TumorMap interface provides an interactive way to explore and navigate through the map, where each sample is represented by a dot localized according to its position in the phenotype space (Fig. 7). The attributes can be used to change colors, filter samples, and perform statistical tests, and new attributes can be derived from preexisting ones using set operations. This flexible and user-friendly interface will enable new hypotheses to be tested without computational expertise and expands the reuse potential of the dataset [55].

## Conclusion

We demonstrated that we provide a high-quality multiomic dataset of malignant pleural mesothelioma, including the largest whole-genome sequencing dataset of malignant pleural mesothelioma to date, consisting of both raw and processed data and important molecular phenotypes. By homogenizing the clinical, epidemiologic, morphologic, and molecular data of our new series with the 2 previously published MPM multiomics data series, we have created an unprecedented dataset for this rare cancer in terms of both size and detail. We provide all the resources to reproduce our analyses, as well as a user-friendly interactive visualization tool, which will contribute to advancing biological knowledge of this deadly disease. As most patients with MPM will survive to second- or third-line systemic therapy, future studies will be needed to describe the molecular landscape of MPM at these time points to develop effective precision medicine strategies.

## Availability of Source Code and Requirements

Project name: MESOMICS data and phenotypic map

Project homepage: https://github.com/IARCbioinfo/MESOMICS_data

Operating system(s): Platform independent

Programming language: R

Other requirements: R packages *data.table, openxlsx, DESeq2, rtracklayer, tibble, IlluminaHumanMethylationEPICanno.ilm10b4.hg19, walaj/roverlaps, reticulate, MOFA2, ParetoTI*.

License: GPL-3.0 license

## Data Availability

The data used in this manuscript are available in the European Genome-Phenome Archive (EGA), which is hosted at the EBI and the Centre for Genomic Regulation (CRG), under the accession number EGAS00001004812; download requires approval from the data access committee EGAC00001001811 (email Dr. Matthieu Foll at follm@iarc.who.int) and then installing the EGA download python client and its dependencies (python3 and pip3; see instructions [56] and a video tutorial [57]). Other data further supporting this work are openly available in the *GigaScience* respository, GigaDB [58].

## Additional Files

**Supplementary Fig. S1**. MPM copy number variant (CNV) cohort profile aCNViewer plot [59] from tumor-matched called as tumor-only (top), tumor-only (middle), and tumor-only after filtering (bottom). The circled regions correspond to artifactual peaks when calling CNVs with the tumor-only mode of PURPLE. The aforementioned genomic regions were identified and filtered, and they are provided in Supplementary Table S1.

**Supplementary Fig. S2**. Correlation between purity estimates from 3 different omic purity measurements: the proportion of DNA material from the tumor (genomic estimate of purity), the complement proportion of infiltrating immune cells (transcriptomic estimate of purity), and the amount of tumor tissue in the observed slide (pathologic estimate of purity). (A) Between transcriptomic and pathologic estimates, (B) between genomic and pathologic estimates, and (C) between genomic and transcriptomic estimates. In these 3 panels, *q* values and coefficient *r* correspond to Pearson correlation tests.

**Supplementary Table S1**. List of excluded genomic regions identified as artifactual when calling CNVs using PURPLE tumor-only mode.

**Supplementary Table S2**. Harmonized and curated molecular, clinical, epidemiologic, and morphologic data from our MESOMICS cohort and the 2 previously published MPM multiomics data [2, 3]. This table can be explored interactively on the UCSC TumorMap web portal.

**Supplementary Note 1**. Additional details of the point mutation and structural variant calling for tumor-only MPM WGS samples.

giac128_GIGA-D-22-00184_Original_Submission

giac128_GIGA-D-22-00184_Revision_1

giac128_GIGA-D-22-00184_Revision_2

giac128_Response_to_Reviewer_Comments_Original_Submission

giac128_Response_to_Reviewer_Comments_Revision_1

giac128_Reviewer_1_Report_Original_Submission Saurabh V Laddha -- 8/23/2022 Reviewed

giac128_Reviewer_2_Report_Original_Submission Jeremy Warner -- 8/31/2022 Reviewed

giac128_Reviewer_3_Report_Original_Submission Mary Ann Tuli -- 9/1/2022 Reviewed

giac128_Supplemental_Files

## Abbreviations

bp: base pair; EGA: European Genome-Phenome Archive; EMBL-EBI: European Bioinformatics Institute; IARC: International Agency for Research on Cancer; MPM: malignant pleural mesothelioma; PCA: principal component analysis; QC: quality control; RF: random forest; RNA-seq: RNA sequencing; STAR: Spliced Transcripts Alignment to a Reference; SV: structural variant; TES: transcription end site; TSS: transcription start site; UCSC: University of California Santa Cruz; vst: variance-stabilized transformation; WGS: whole-genome sequencing.

## Ethics Approval

These data belong to the MESOMICS project, which has been approved by the IARC Ethical Committee.

## Competing Interests

Where authors are identified as personnel of the International Agency for Research on Cancer/World Health Organization, the authors alone are responsible for the views expressed in this article and they do not necessarily represent the decisions, policy, or views of the International Agency for Research on Cancer/World Health Organization.

## Funding

This work has been funded by the French National Cancer Institute (INCa, PRT-K 2016–039 to L.F.C. and M.F.) and the Ligue Nationale contre le Cancer (LNCC 2017 and 2020 to L.F.C. and M.F.). L.M. has a fellowship from the LNCC.

## Authors' Contributions

Conceptualization: M.F., N.A., L.F.-C. Methodology: M.F., N.A., L.F.-C., L.M., A.D.G., A.S.-O. Software: A.D.G., L.M., A.S.-O, C.V., N.A. Validation: A.D.G., L.M., A.S.-O, N.A. Formal analyses: A.D.G., L.M., A.S.-O, C.V., N.A. Investigation: A.D.G., L.M., A.S.-O, C.V., N.A. Data curation: A.D.G., L.M., A.S.-O, C.V., N.A. Writing—original draft: A.D.G., M.F., L.M., N.A., A.S.-O. Writing—review & editing: A.D.G., M.F., L.M., N.A., A.S.-O., L.F.-C. Visualization: A.D.G, L.M., N.A., A.S.-O., C.V. Supervision: L.F.-C., M.F., N.A. Project administration: L.F.-C., M.F., L.M., N.A. Funding acquisition: L.F.-C., M.F., N.A.

## References

[1] WHO Classification of Tumours Editorial Board . Thoracic Tumours: WHO Classification of Tumours. 5th ed. Geneva, Switzerland: WHO; 2021.

[2] Bueno R, Stawiski EW, Goldstein LD, et al. Comprehensive genomic analysis of malignant pleural mesothelioma identifies recurrent mutations, gene fusions and splicing alterations. Nat Genet. 2016;48:407–16. 26928227 10.1038/ng.3520

[3] Hmeljak J, Sanchez-Vega F, Hoadley KA et al. Integrative molecular characterization of malignant pleural mesothelioma. Cancer Discov. 2018;8:1548–65. 30322867 10.1158/2159-8290.CD-18-0804 PMC6310008

[4] Alcala N, Mangiante L, Le-Stang N, et al. Redefining malignant pleural mesothelioma types as a continuum uncovers immune-vascular interactions. EBioMedicine. 2019;48:191–202. 31648983 10.1016/j.ebiom.2019.09.003 PMC6838392

[5] Blum Y, Meiller C, Quetel L, et al. Dissecting heterogeneity in malignant pleural mesothelioma through histo-molecular gradients for clinical applications. Nat Commun. 2019;10:1333. 30902996 10.1038/s41467-019-09307-6 PMC6430832

[6] Fernandez-Cuesta L, Mangiante L, Alcala N, et al. Challenges in lung and thoracic pathology: molecular advances in the classification of pleural mesotheliomas. Virchows Arch. 2021;478:73–80. 33411030 10.1007/s00428-020-02980-9

[7] Cortés-Ciriano I, Lee JJ-K, Xi R, et al. Comprehensive analysis of chromothripsis in 2,658 human cancers using whole-genome sequencing. Nat Genet. 2020;52:331–41. 32025003 10.1038/s41588-019-0576-7 PMC7058534

[8] Kim H, Nguyen N-P, Turner K, et al. Extrachromosomal DNA is associated with oncogene amplification and poor outcome across multiple cancers. Nat Genet. 2020;52:891–7. 32807987 10.1038/s41588-020-0678-2 PMC7484012

[9] Quinton RJ, DiDomizio A, Vittoria MA, et al. Whole-genome doubling confers unique genetic vulnerabilities on tumour cells. Nature. 2021;590:492–7. 33505027 10.1038/s41586-020-03133-3 PMC7889737

[10] Mangiante L, Alcala N, Di Genova A, et al. Multi-omic analysis of malignant pleural mesothelioma identifies molecular axes and specialized tumor profiles driving inter-tumor heterogeneity. Nat Genet. In press. 10.1038/s41588-023-01321-1 PMC10101853 36928603

[11] Hausser J, Alon U. Tumour heterogeneity and the evolutionary trade-offs of cancer. Nat Rev Cancer. 2020;20:247–57. 32094544 10.1038/s41568-020-0241-6

[12] Newton Y, Novak AM, Swatloski T, et al. TumorMap: exploring the molecular similarities of cancer samples in an interactive portal. Cancer Res. 2017;77:e111–4. 29092953 10.1158/0008-5472.CAN-17-0580 PMC5751940

[13] IARCbioinfo/MESOMICS_data GitHub repository. https://github.com/IARCbioinfo/MESOMICS_data, (last accessed date: 5 July 2022).

[14] Zalcman G, Mazieres J, Margery J, et al. Bevacizumab for newly diagnosed pleural mesothelioma in the Mesothelioma Avastin Cisplatin Pemetrexed Study (MAPS): a randomised, controlled, open-label, phase 3 trial. Lancet North Am Ed. 2016;387:1405–14. 10.1016/S0140-6736(15)01238-6 26719230

[15] Baas P, Scherpereel A, Nowak AK, et al. First-line nivolumab plus ipilimumab in unresectable malignant pleural mesothelioma (CheckMate 743): a multicentre, randomised, open-label, phase 3 trial. Lancet North Am Ed. 2021;397:375–86. 10.1016/S0140-6736(20)32714-8 33485464

[16] Di Tommaso P, Chatzou M, Floden EW, et al. Nextflow enables reproducible computational workflows. Nat Biotechnol. 2017;35:316–9. 28398311 10.1038/nbt.3820

[17] IARCbioinfo/fastqc-nf GitHub repository. https://github.com/IARCbioinfo/fastqc-nf, (last accessed date: 12 January 2020).

[18] IARCbioinfo/alignment-nf Github repository. https://github.com/IARCbioinfo/alignment-nf, (last accessed date: 12 January 2020).

[19] IARCbioinfo/RNAseq-nf Github repository. https://github.com/IARCbioinfo/RNAseq-nf, (last accessed date: 2 June 2020).

[20] Gabriel AAG, Mathian E, Mangiante L, et al. A molecular map of lung neuroendocrine neoplasms. Gigascience. 2020;9:giaa112. 10.1093/gigascience/giaa112 PMC7596803 33124659

[21] Dobin A, Davis CA, Schlesinger F, et al. STAR: ultrafast universal RNA-seq aligner. Bioinformatics. 2013;29:15–21. 23104886 10.1093/bioinformatics/bts635 PMC3530905

[22] IARCbioinfo/abra-nf Github repository. https://github.com/IARCbioinfo/abra-nf, (last accessed date: 2 June 2020).

[23] Mose LE, Perou CM, Parker JS. Improved indel detection in DNA and RNA via realignment with ABRA2. Bioinformatics. 2019;35:2966–73. 30649250 10.1093/bioinformatics/btz033 PMC6735753

[24] IARCbioinfo/BQSR-nf Github repository. https://github.com/IARCbioinfo/BQSR-nf, (last accessed date: 2 June 2020).

[25] Van der Auwera GA, O'Connor BD. Genomics in the Cloud: Using Docker, GATK, and WDL in Terra. Sebastopol, CA (USA): O'Reilly Media, Inc; 2020.

[26] IARCbioinfo/RNAseq-transcript-nf Github repository. https://github.com/IARCbioinfo/RNAseq-transcript-nf, (last accessed date: 2 June 2020).

[27] Wang L, Wang S, Li W. RSeQC: quality control of RNA-seq experiments. Bioinformatics. 2012;28:2184–5. 22743226 10.1093/bioinformatics/bts356

[28] Ewels P, Magnusson M, Lundin S, et al. MultiQC: summarize analysis results for multiple tools and samples in a single report. Bioinformatics. 2016;32:3047–8. 27312411 10.1093/bioinformatics/btw354 PMC5039924

[29] IARCbioinfo/Methylation_analysis_scripts Github repository. https://github.com/IARCbioinfo/Methylation_analysis_scripts, (last accessed date: 2 June 2020).

[30] IARCbioinfo/purple-nf Github repository. https://github.com/IARCbioinfo/purple-nf, (last accessed date: May 2021).

[31] Priestley P, Baber J, Lolkema MP, et al. Pan-cancer whole-genome analyses of metastatic solid tumours. Nature. 2019;575:210–6. 31645765 10.1038/s41586-019-1689-y PMC6872491

[32] Cameron DL, Baber J, Shale C, et al. GRIDSS, PURPLE, LINX: unscrambling the tumor genome via integrated analysis of structural variation and copy number. bioRxiv. 781013. 10.1101/781013. PMC9903802 36776527

[33] hartwigmedical/hmftools Github issue. https://github.com/hartwigmedical/hmftools/issues/102, (last accessed date: 9 May 2021).

[34] Liaw A, Wiener M. Classification and regression by random forest. R News. 2002;2:18–22.

[35] IARCbioinfo/mutect-nf Github repository. https://github.com/IARCbioinfo/mutect-nf, (last accessed date: 6 October 2020).

[36] Karczewski KJ, Francioli LC, Tiao G, et al. The mutational constraint spectrum quantified from variation in 141,456 humans. Nature. 2020;581:434–43. 32461654 10.1038/s41586-020-2308-7 PMC7334197

[37] Alexandrov LB, Kim J, Haradhvala NJ, et al. The repertoire of mutational signatures in human cancer. Nature. 2020;578:94–101. 32025018 10.1038/s41586-020-1943-3 PMC7054213

[38] Danecek P, Bonfield JK, Liddle J, et al. Twelve years of SAMtools and BCFtools. Gigascience. 2021;10:giab008. 10.1093/gigascience/giab008 PMC7931819 33590861

[39] Wala JA, Bandopadhayay P, Greenwald NF, et al. SvABA: genome-wide detection of structural variants and indels by local assembly. Genome Res. 2018;28:581–91. 29535149 10.1101/gr.221028.117 PMC5880247

[40] Chen X, Schulz-Trieglaff O, Shaw R, et al. Manta: rapid detection of structural variants and indels for germline and cancer sequencing applications. Bioinformatics. 2016;32:1220–2. 26647377 10.1093/bioinformatics/btv710

[41] Rausch T, Zichner T, Schlattl A, et al. DELLY: structural variant discovery by integrated paired-end and split-read analysis. Bioinformatics. 2012;28:i333–9. 22962449 10.1093/bioinformatics/bts378 PMC3436805

[42] Jeffares DC, Jolly C, Hoti M, et al. Transient structural variations have strong effects on quantitative traits and reproductive isolation in fission yeast. Nat Commun. 2017;8:14061. 28117401 10.1038/ncomms14061 PMC5286201

[43] IARCbioinfo/sv_somatic_cns-nf Github repository. https://github.com/IARCbioinfo/sv_somatic_cns-nf, (last accessed date: 3 November 2021).

[44] Li Y, Roberts ND, Wala JA, et al. Patterns of somatic structural variation in human cancer genomes. Nature. 2020;578:112–21. 32025012 10.1038/s41586-019-1913-9 PMC7025897

[45] Pollard KS, Hubisz MJ, Rosenbloom KR, et al. Detection of nonneutral substitution rates on mammalian phylogenies. Genome Res. 2010;20:110–21. 19858363 10.1101/gr.097857.109 PMC2798823

[46] IARCbioinfo/RF-mut-f Github repository. https://github.com/IARCbioinfo/RF-mut-f, (last accessed date: 3 November 2021).

[47] IARCbioinfo/ssvht Github repository. https://github.com/IARCbioinfo/ssvht, (last accessed date: 3 November 2021).

[48] Lee S, Lee S, Ouellette S et al. NGSCheckMate: software for validating sample identity in next-generation sequencing studies within and across data types. Nucleic Acids Res. 2017;45:e103. 28369524 10.1093/nar/gkx193 PMC5499645

[49] IARCbioinfo/NGSCheckMate-nf Github repository. https://github.com/IARCbioinfo/NGSCheckMate-nf, (last accessed date: 12 July 2021).

[50] Finotello F, Mayer C, Plattner C, et al. Molecular and pharmacological modulators of the tumor immune contexture revealed by deconvolution of RNA-seq data. Genome Med. 2019;11:34. 31126321 10.1186/s13073-019-0638-6 PMC6534875

[51] Argelaguet R, Velten B, Arnol D et al. Multi-omics factor analysis—a framework for unsupervised integration of multi-omics data sets. Mol Syst Biol. 2018;14:e8124. 29925568 10.15252/msb.20178124 PMC6010767

[52] Kleshchevnikov V . ParetoTI R package. Zenodo 2019. doi:10.5281/zenodo.2853581.

[53] Hausser J, Szekely P, Bar N, et al. Tumor diversity and the trade-off between universal cancer tasks. Nat Commun. 2019;10:1–13. 31780652 10.1038/s41467-019-13195-1 PMC6882839

[54] Mak MP, Tong P, Diao L, et al. A patient-derived, pan-cancer EMT signature identifies global molecular alterations and immune target enrichment following epithelial-to-mesenchymal transition. Clin Cancer Res. 2016;22:609–20. 26420858 10.1158/1078-0432.CCR-15-0876 PMC4737991

[55] MESOMICS TumorMap. https://tumormap.ucsc.edu/?p=RCG_MESOMICS/MPM_Archetypes, (last accessed date: 15 November 2022).

[56] EGA download client. https://github.com/EGA-archive/ega-download-client, (last accessed date: 15 November 2022).

[57] EGA download tutorial. https://embl-ebi.cloud.panopto.eu/Panopto/Pages/Viewer.aspx?id=be79bb93-1737-4f95-b80f-ab4300aa6f5a, (last accessed date: 15 November 2022).

[58] Di Genova A, Mangiante L, Sexton-Oates A, et al. Supporting data for “A molecular phenotypic map of malignant pleural mesothelioma.” GigaScience Database. 2022. 10.5524/102342. PMC9881451 36705549

[59] Renault V, Tost J, Pichon F, et al. aCNViewer: comprehensive genome-wide visualization of absolute copy number and copy neutral variations. PLoS One. 2017;12:e0189334. 29261730 10.1371/journal.pone.0189334 PMC5736239

