# Supplementary material for: A molecular phenotypic map of malignant pleural mesothelioma
Source: Gigascience. 2023 Jan 27;12:giac128. doi: 10.1093/gigascience/giac128 (PMC9881451; doi:10.1093/gigascience/giac128)
Supplement: giac128_GIGA-D-22-00184_Revision_1 [file giac128_giga-d-22-00184_revision_1.pdf]

GigaScience

A molecular phenotypic map of Malignant Pleural Mesothelioma

--Manuscript Draft--

**Manuscript Number:**	GIGA-D-22-00184R1
**Full Title:**	A molecular phenotypic map of Malignant Pleural Mesothelioma
**Article Type:**	Data Note
**Funding Information:**	Institut National Du Cancer (PRT-K 2016-039) — Dr Lynnette Fernandez-Cuesta, Dr Matthieu Foll<br>Ligue Contre le Cancer — Dr Lynnette Fernandez-Cuesta, Dr Matthieu Foll
**Abstract:**	Background: Malignant Pleural Mesothelioma (MPM) is a rare understudied cancer associated with exposure to asbestos. So far, MPM patients have benefited marginally from the genomics medicine revolution due to the limited size or breadth of existing molecular studies. In the context of the MESOMICS project, we have performed the most comprehensive molecular characterization of MPM to date, with the underlying dataset made of the largest whole genome sequencing series yet reported, together with transcriptome sequencing and methylation arrays for 120 MPM patients.<br>Results: We first provide comprehensive quality controls for all samples, of both raw and processed data. Due to the difficulty in collecting specimens from such rare tumors, a part of the cohort does not include matched normal material. We provide a detailed analysis of data processing of these tumor-only samples, showing that all somatic alteration calls match very stringent criteria of precision and recall. Finally, integrating our data with previously published multi-omic MPM datasets (n=374 in total), we provide an extensive molecular phenotype map of MPM based on the multi-task theory. The generated map can be interactively explored and interrogated on the UCSC TumorMap portal (https://tumormap.ucsc.edu/RCG_MESOMICS/MPM_Archetypes).<br>Conclusions: This new high quality MPM multi-omics dataset, together with the state-of-art bioinformatics and interactive visualization tools we provide, will support the development of precision medicine in MPM that is particularly challenging to implement in rare cancers due to limited molecular studies.
**Corresponding Author:**	Matthieu Foll<br><br>FRANCE
**Corresponding Author Secondary Information:**	
**Corresponding Author's Institution:**	
**Corresponding Author's Secondary Institution:**	
**First Author:**	Alex Di Genova
**First Author Secondary Information:**	
**Order of Authors:**	Alex Di Genova
	Lise Mangiante
	Alexandra Sexton-Oates
	Catherine Voegele
	Lynnette Fernandez-Cuesta
	Nicolas Alcala
	Matthieu Foll
**Order of Authors Secondary Information:**	

Powered by Editorial Manager® and ProduXion Manager® from Aries Systems Corporation

Response to Reviewers:	Dear Dr Nogoy,<br><br>We would like to thank you for the prompt and thorough review of our article entitled "A molecular phenotypic map of Malignant Pleural Mesothelioma", and for giving us the possibility of addressing the reviewers' comments. We have now generated a revised version of the manuscript taking into account all suggestions. We have also addressed the editor's and reviewers' comments point-by-point, below. We have highlighted changes in the main text in yellow for easier reference.<br><br>Please note that this Data Note is aimed to be published in coordination with or after the publication of our analysis paper, which has just been accepted in Nature Genetics. We apologize it took us more time than we expected to revise the manuscript because we had to work on both papers in parallel.<br><br>We very much look forward to your consideration of our revised manuscript for publication in GigaScience.<br><br>Best regards,<br><br>Matthieu<br><br>Reviewer #1:<br><br>Authors did a fantastic job by integrating MPM multi-omics datasets and created an integrative and interactive map for users to explore these datasets. MPM is a rare cancer type and understudied so such resources are very useful to move the field forward at a molecular level. The comprehensive data is well presented and the manuscript is well written to explain the complex genomics dataset for MPM.<br>All the figures are well explained and very clear to understand<br><br>Answer: We thank the reviewer for his enthusiasm, the useful comments, for noting the value of our work and how it will contribute to move forward MPM research.<br><br>Minor point:<br>- Author mentioned an evaluation of tumor purity was done using pathological review, did author used molecular data such as genomic data to find tumor purity ? and if yes, how was the consensus ? This is very important factor to interpret the genomic results as the data was sequenced at 30X<br>- In the same line, RNAseq can also be used to identify tumor purity and it will be really helpful for users to clear picture on tumor purity.<br><br>Answer: We now report purity estimates from genomic and expression data in Table S2, and show their correlation with the pathological review estimates in a new "Purity" subsection of "Data Validation" p. 11.<br><br>- Is it not very clear from method section that the same MPM samples were used to sequence at DNA , RNA and DNA methylation level ? A brief explanation or table will be very easy for users to understand.<br><br>Answer: We now mention in the text (beginning of Data Description section p. 3) that a vast majority of the samples (105/120) had complete data (WGS, RNA-seq and methylation), and provide in Table S2 the list of samples with each omic data available.<br><br>- Recent WHO classify MPM into three different histopathological types. Did author do any unsupervised analysis from these comprehensive data to understand MPM heterogeneity or replicate WHO classification? or did author find WHO subtypes of MPM using molecular dataset ? A brief analysis/comment on usage of histological classification Vs Molecular classification will certainly move the MPM research field forward as researcher have found vast differences between histological vs molecular classification and the field is moving towards more molecular based classification in clinic.<br><br>Answer: This paper is a Data Note to be published in coordination with or after the publication of our analysis paper, in which we indeed performed these in depth

Powered by Editorial Manager® and ProduXion Manager® from Aries Systems Corporation

analyses (Mangiante et al. 2021, preprint available at https://www.biorxiv.org/content/10.1101/2021.09.27.461908v1, ref 10 in the manuscript, now accepted for publication in Nature Genetics). We apologize this was unclear, and we have now added a sentence at the beginning of the Data Description (p. 4) section referring to this paper.

Reviewer #2:

In this paper, the authors describe a new public resource for the molecular characterization of malignant pleural mesothelioma (MPM), which they describe as the most comprehensive to date. They perform WGS, transcriptome, and methylation arrays for 120 patients with MPM sourced through the MESOMICS project and integrate this dataset with an additional several hundred patients from previously published datasets.

Although I cannot independently verify their claim that this is the largest and most comprehensive dataset for MPM, it is quite impressive and expansive. The pipeline utilized is well described and the results at all stages are transparently shared for prospective users of this dataset.

Answer: We thank the reviewer for his/her review, and in particular for noting the importance of transparent sharing of the data processing and analysis.

The description of the methods to identify and remove germline variants is interesting, although the length somewhat detracts from the main goal of the paper in describing an MPM resource. Perhaps, this part could be condensed with the technical details presented in supplement. This comment pertains to both the Point Mutations and Structural Variants sections.

Answer: We have followed the reviewer suggestion and have condensed (from 1610 to 757 words) this part of the methods and put the more technical details in the supplementary Note 1.

Additional moderate concerns:

There are insufficient details provided on the clinical and epidemiological parameters. Indirectly, it would appear that sex, age class, and smoking status are the clinical parameters - but what are the age classes? Is smoking status binary ever/never, or more involved? There ought to be a data dictionary provided as a supplemental table which describes each clinical/epidemiological variable, along with the possible values that the variable can take on. It should additionally be explained why other important clinical parameters are not available - most importantly, the presence of accompanying pulmonary comorbidity such as chronic obstructive pulmonary disease (COPD) and the existence of conditions that might preclude the use of standard systemic therapies, such as renal disease precluding the use of platinum agents.

Answer: We apologize for the lack of clarity surrounding the clinical data and now provide more details about the clinical and epidemiological characteristics of the cohort. We have added a data dictionary for Table S2 (2nd worksheet tab in the Excel file), and now mention it on p. 4. We also explicitly state which clinical data is available on p. 4, in particular mentioning that we “provide basic clinical data (age, sex, survival) as well as exposure (asbestos, smoking), and treatment data (usage and type of chemotherapy, surgery, radiotherapy, and precision treatment),” and that “Comorbidity data were not available, however we provide symptoms that are informative on the state of the patient at diagnosis (pain, pleural effusion, dyspnea, pneumothorax, coughing).”

Context: I would like to see more here about the role of asbestos in the etiology, including what might be known about the pathophysiology of asbestos fibers at the molecular level. Also, there is nothing here about the evolution of treatment for MPM; the latest "state-of-the-art" regimens (platinum doublet + bevacizumab [MAPS; NCT00651456] and dual checkpoint inhibition [Checkmate 743; NCT02899299]) have reported median survival in the 18-month range, which is distinctly better than the median survivals quoted by the authors. Finally, I would like to see one or more direct

Powered by Editorial Manager® and ProduXion Manager® from Aries Systems Corporation

references to the clinical trials where molecular heterogeneity has "fueled the implementation of drug trials for more tailored MPM treatments".

Answer: This paper is a Data Note to be published in coordination with or after the publication of our analysis paper, in which we discuss the effect of asbestos fiber at the molecular level (Mangiante et al. 2021, preprint available at https://www.biorxiv.org/content/10.1101/2021.09.27.461908v1, ref 10 in the manuscript, now accepted for publication in Nature Genetics). We apologize this was unclear, and we have now added a sentence at the beginning of the Data Description (p. 4) section referring to this paper.

We now mention p. 4 that a vast majority of patients were treated with chemotherapy, surgery, or radiotherapy and a single patient received immunotherapy (now in Table S2). We also mention that because of the retrospective nature of the samples from the French MESOBANK, patients were diagnosed (year of diagnosis [1998-2017], median of 2011) and treated (year of death or end of follow-up [2000-2020], median of 2013) before the results from the MAPS (2016) and Checkmate 743 trials (2021), and before the authorization of nivolumab and ipilimumab by the European Medicines Agency in 2022 (note that despite the MAPS trial, bevacizumab is not a standard first line treatment in France); we thus indicate to the readers that future studies will hopefully report longer survivals as the reviewer mentions, and cite these clinical trials.

Data Description: All specimens in the MESOMICS study are said to be collected from surgically resected MPM; this also appears to be the case for the integrated multi-omic studies from Bueno et al. and Hmeljak et al. and this should be explicitly indicated. Somewhere, it should also be explicitly discussed that this is an important limitation in the future utility of this data - surgical specimens are convenience samples and while they do provide important information, they lack treatment exposure. Given that many if not most patients with MPM will survive to 2nd or 3rd line systemic therapy, and that 1st line is fairly standardized, a knowledge of induced mutations is going to be essential to the ultimate goal of precision medicine.

Answer: We thank the reviewer for this important comment. All specimens in the MESOMICS study are collected from surgically resected MPM, this is also the case for the majority of samples from the Bueno et al. and Hmeljak et al studies. Specifically, in Hmeljak et al. frozen primary tumors were obtained from surgical resection (n=55), biopsy (n=9), or unknown surgery type (n=10), and in Bueno et al. fresh-frozen samples were obtained from patients undergoing extirpative surgery for malignant pleural mesothelioma. This information has now been reported on p. 12 and added to Table S2. We have also added the important point that future studies will be needed to describe the molecular landscape of MPM after 1st and 2nd line of systemic therapy to develop effective precision medicine strategies (final conclusion sentence of the manuscript p. 14).

Minor concerns:

The labels in the figures (e.g., Figure 2 - "Unmapped..too.short") are human-readable but could still be presented in a more friendly fashion. All acronyms should be defined.

Answer: We have updated the labels by removing the dots and underscores, thanks for spotting this error.

Reviewer #3:

I am reviewing only process of obtaining access to the controlled data described in your study.
You have been very prompt and clear in all communication regarding the DAA and access to EGAS00001004812 was promptly granted by the EGA.
As you are aware I am having difficulty actually downloading the 3 datasets, but I am confident the issues will be resolved soon.

Answer: We thank you for carefully going through the entire data access process to ensure that the data is available. We now provide more information about the process of requesting access and setting up the download of the data p. 14.

Additional Information:	
Question	Response
Are you submitting this manuscript to a special series or article collection?	No
**Experimental design and statistics**<br><br>Full details of the experimental design and statistical methods used should be given in the Methods section, as detailed in our Minimum Standards Reporting Checklist. Information essential to interpreting the data presented should be made available in the figure legends.<br><br>Have you included all the information requested in your manuscript?	Yes
**Resources**<br><br>A description of all resources used, including antibodies, cell lines, animals and software tools, with enough information to allow them to be uniquely identified, should be included in the Methods section. Authors are strongly encouraged to cite Research Resource Identifiers (RRIDs) for antibodies, model organisms and tools, where possible.<br><br>Have you included the information requested as detailed in our Minimum Standards Reporting Checklist?	Yes
**Availability of data and materials**<br><br>All datasets and code on which the conclusions of the paper rely must be either included in your submission or deposited in publicly available repositories (where available and ethically appropriate), referencing such data using a unique identifier in the references and in the "Availability of Data and Materials" section of your manuscript.	Yes

Have you have met the above requirement as detailed in our Minimum Standards Reporting Checklist?	

Manuscript

Click here to access/download;Manuscript;MainText_changes_highlighted.d

A molecular phenotypic map of Malignant Pleural Mesothelioma

Alex Di Genova[1,2,2b,#], Lise Mangiante[1,1b,#], Alexandra Sexton-Oates[1], Catherine Voegele[1], Lynnette Fernandez-Cuesta[1,†], Nicolas Alcala[1,†], Matthieu Foll[1,†]

[#]These authors contributed equally to this work

[†]These authors jointly supervised this work

[1]Rare Cancers Genomics Team (RCG), Genomic Epidemiology Branch (GEM), International Agency for Research on Cancer/World Health Organisation (IARC/WHO), Lyon, 69008, France

[1b]Department of Medicine, Stanford University, Stanford, USA

[2]Instituto de Ciencias de la Ingeniería, Universidad de O'Higgins, Rancagua, Chile

[2b]Centro de Modelamiento Matemático UMI-CNRS 2807, Universidad de Chile, Santiago, Chile

Correspondence should be addressed to MF (follm@iarc.who.int)

Abstract

Background Malignant Pleural Mesothelioma (MPM) is a rare understudied cancer associated with exposure to asbestos. So far, MPM patients have benefited marginally from the genomics medicine revolution due to the limited size or breadth of existing molecular studies. In the context of the MESOMICS project, we have performed the most comprehensive molecular characterization of MPM to date, with the underlying dataset made of the largest whole genome sequencing series yet reported, together with transcriptome sequencing and methylation arrays for 120 MPM patients. **Results** We first provide comprehensive quality controls for all samples, of both raw and processed data. Due to the difficulty in collecting specimens from such rare tumors, a part of the cohort does not include matched normal material. We provide a detailed analysis of data processing of these tumor-only samples, showing that all somatic alteration calls match very stringent criteria of precision and recall. Finally, integrating our data with previously published multi-omic MPM datasets (*n=374* in total), we provide an extensive molecular phenotype map of MPM based on the multi-task theory. The generated map can be interactively explored and interrogated on the UCSC TumorMap portal (https://tumormap.ucsc.edu/RCG_MESOMICS/MPM_Archetypes). **Conclusions** This new high quality MPM multi-omics dataset, together with the state-of-art bioinformatics and interactive visualization tools we provide, will support the development of precision medicine in MPM that is particularly challenging to implement in rare cancers due to limited molecular studies.

Key words: Malignant Pleural Mesothelioma, genomics, transcriptomics, DNA methylation, quality control, cancer tasks, tumor map.

Context

Malignant Pleural Mesothelioma (MPM) is a deadly pleural cancer with currently limited therapeutic opportunities that translate into poor outcomes for patients. The latest WHO classification [1] recognises three different histopathological types, namely epithelioid (MME, median overall survival of 14.4 months), biphasic (MMB, 9.5 months), and sarcomatoid (MMS, 5.3 months). Multi-omic sequencing data [2,3] have been key in the identification of driver genes, developing and refining the characterisation of molecular profiles from initial discrete clusters to a continuum [4–6], and uncovering rare genotypes such as near-haploid genomes. Such advances have revealed the rich molecular heterogeneity in MPM, and have fueled the implementation of drug trials for more tailored MPM treatments. Despite their important findings, these multi-omic studies have profiled only a reduced representation of the MPM genome (primarily exomes) and have mainly focused on describing simple mutational processes (i.e. copy number alterations and point mutations). Therefore, there is still a need for comprehensive multi-omic datasets including whole MPM genome sequences to allow the study of complex mutational processes–e.g., whole-genome doubling (WGD), chromothripsis, extrachromosomal DNA (ecDNA)–that have been described in other cancer types [7–9] but not in MPM. Furthermore, understanding how genomic events impact tumor phenotypes remains poorly studied in MPM. Finally, given that MPM is a rare disease, the integration of different multi-omic studies is essential for reaching the statistical power needed to derive insightful biological conclusions from complex multi-omic datasets.

Data Description

Here we describe the dataset generated by the MESOMICS project that collected more than one hundred MPM tumors with extensive clinical, epidemiological, and morphological annotations, and profiled their genome, transcriptome, and epigenome. Notably, MESOMICS prioritized the sequencing of whole MPM genomes rather than exomes, resulting in the largest set of MPM genome sequences available to date. In total, we sequenced 120 MPM tumors, among which a vast majority (105) have the three omic' data available, and the remaining 15 samples have one or two omic' data types (**Supplementary Table S2**). This dataset has been deposited at the EMBL-EBI European Genome-phenome Archive (EGA accession No. EGAS00001004812), and has been used to propose a new morpho-molecular classification of MPM [10]. Here, we provide a comprehensive description of data quality control, and links to all bioinformatic pipelines used in the project, including state-of-the-art methodology for mutational calling in tumor-only specimens. Finally, in order to maximize the reuse potential of our MESOMICS data, we integrate our cohort with the previous multi-omic studies from Bueno *et al.* [2] and Hmeljak *et al.* [3] to generate the first multi-cohort molecular phenotypic map for MPM based on the multi-task Pareto optimum theory [11]. This interactive map provides a user-friendly way to explore the

molecular data and to generate new hypotheses through custom statistical tests, based on the UCSC TumorMap portal [12]. The integrated and harmonized dataset resulting from these studies is available on GitHub [13].

Primary tumor specimens were collected from surgically resected MPM. As described in Mangiante *et al.* 2021 [10], among them 13 had two tumor specimens collected to study intratumoral heterogeneity; we report quality controls for all samples including these 13 additional samples, but only the piece with the highest tumor content as estimated by pathological review was selected for subsequent analyses, except for analyses that specifically focused on intra-tumor heterogeneity. The samples used in this study belong to the French MESOBANK. Our pathologist (FGS) classified all tumors following the latest WHO guidance and DNA, and RNA extraction methods are described in the methods section of our recent study [10].

We provide basic clinical data (age, sex, survival) as well as exposure (asbestos, smoking), and treatment data (usage and type of chemotherapy, surgery, radiotherapy, and precision treatment) (see detailed data dictionary in **Supplementary Table S2**). Comorbidity data were not available, however we provide where available symptoms reported at diagnosis that are informative on the state of the patient at diagnosis (pain, pleural effusion, dyspnea, pneumothorax, coughing). Note that because of the retrospective nature of the samples from the French MESOBANK, patients were diagnosed (year of diagnosis [1998-2017], median of 2011) and treated (year of death or end of follow-up [2000-2020], median of 2013) before the results of recent promising clinical trials (MAPS [14] and Checkmate 743 [15]), and before the authorization of nivolumab and ipilimumab by the European Medicines Agency in 2022 (note that despite the MAPS trial, bevacizumab is not a standard first line treatment in France); future studies will thus probably include more patients who underwent precision treatments and hopefully report longer survivals [15].

Quality control of omic data

Whole-Genome sequencing (WGS)

Whole-genome sequencing was performed by the Centre National de Recherche en Génomique Humaine (CNRGH, Institut de Biologie François Jacob, CEA, Evry, France) on 130 fresh frozen MPMs, plus 54 matched-normal tissue or blood samples (matched non-neoplastic tissue was not available for the other specimens). The Illumina TruSeq DNA PCR-Free Library Preparation Kit was used for library preparation and the HiSeqX5 platform from Illumina for the sequencing as described in [10]. The raw WGS reads were scanned by the FastQC software (v.0.11.5; RRID:SCR_014583; using our nextflow pipeline https://github.com/IARCbioinfo/fastqc-nf) to determine the reads base quality, adapter content and duplication levels. The software MultiQC (v0.9; RRID:SCR_014982) was then used to aggregate all the FastQC reports across samples.

The target read output for matched-normal tissue or blood (hereinafter called "matched-normal") and for tumor tissues without matched-normal sample (hereinafter called "tumor-only") was 900M reads (~30X genome coverage, **Figure 1A**). 1800M (~60X genome coverage, **Figure 1A**) were expected for tumor tissues with matched-normal samples (two sequencing lanes, hereinafter called "tumor-matched"). Overall, the median number of reads obtained approached or exceeded the target read output, with median and standard deviation by sample type equal to: matched-normal 889±50, matched-tumor 1786±163, and tumor-only 853±51 million reads (**Figure 1A**).

All samples displayed the expected mean quality score (30Q > 85% of bases) across all base positions of the read (**Figure 1B**). One exception is the MESO_050_N (a matched-normal sample) that on average had a good sequence quality score (**Figure 1B**) but displayed a low mean quality score for the first nucleotide of the read (24.08 Phred), which FastQC reported as a warning in the mean quality score module (**Figure 1B**). In fact, the FastQC report for this sample indicated that 25.17% of bases were not called at the first nucleotide of the read, suggesting that the base-calling process struggled in interpreting the DNA bases at this position and put an N instead. However, the reverse pair-end file of this sample had the expected sequence quality score over all the read positions (**Figure 1B**) and we decided then to include this sample in the subsequent analyses. The adapter content was lower than 1% for all sequenced samples (maximum 0.87% of total reads). The relative level of duplication found for every sequence per sample was on average 10.3% (min: 0% and max: 18.2%), this low level of duplication indicates that the prepared genomic libraries were diverse and likely covered a high proportion of the human genome.

Paired-end read mapping was performed with our nextflow [16] pipeline alignment-nf (v1.0, https://github.com/IARCbioinfo/alignment-nf). This pipeline includes the software qualimap (v2.2.2b; RRID:SCR_001209) and MultiQC to generate comprehensive QC statistics reports from the WGS alignment files. The mean percentage of aligned reads was 98.93±0.81% (**Figure 1C**). The matched-normal and tumor-only samples displayed a mean genome coverage higher than 30X (**Figure 1D**). The matched-tumor displayed a mean genome coverage of 60X (**Figure 1D**). Finally, 90% of the reference genome was covered by at least 22, 20, and 43 reads for matched-normal, tumor-only, and matched-tumor samples, respectively (**Figure 1D**).

RNA Sequencing data

RNA sequencing was performed on 126 fresh frozen MPM in the Cologne Center for Genomics. Libraries were prepared using the Illumina® TruSeq® RNA sample preparation Kit, the Illumina TruSeq PE Cluster Kit v3, and an Illumina TruSeq SBS Kit v3-HS subsequent sequencing was carried out in an Illumina HiSeq 2000 sequencer, as described in [10].

The resulting raw reads files were processed using our nextflow RNA-seq processing pipeline (https://github.com/IARCbioinfo/RNAseq-nf v2.3), as described previously [10,17], that performs reads trimming (Trim Galore v0.6.5; RRID:SCR_011847), and mapping to reference genome GRCh38 (gencode version 33) with STAR (v2.7.3a; RRID:SCR_004463) [18]. We also improve the alignments as described previously by performing assembly based realignment (nextflow pipeline https://github.com/IARCbioinfo/abra-nf, v3.0), using software ABRA2 [19] (RRID:SCR_003277) and base quality score recalibration (nextflow pipeline https://github.com/IARCbioinfo/BQSR-nf, v1.1), using GATK v4.1.7.0 [20] (RRID:SCR_001876). Gene-level quantification was performed using software StringTie (v2.1.2; RRID:SCR_016323) (nextflow pipeline https://github.com/IARCbioinfo/RNAseq-transcript-nf, v2.2). Quality control of the samples was performed using FastQC (v0.11.9; RRID:SCR_014583) to determine the quality of the raw reads, followed by RSeQC (v3.0.1; RRID:SCR_005275) [21] that was used to determine the alignment quality and distribution of reads over the reference genome (number of mapped reads, proportion of uniquely mapped reads). Finally, the software MultiQC (v0.9; RRID:SCR_014982) [22] was used to aggregate all QC results across samples.

A total of 126 samples were sequenced using 2x75bp or 2x100bp pair-end reads (**Figure 2A**). On average, a total of 64±7.4 paired-end million reads were generated with a per sequence mean quality score higher than 35 (**Figure 2A**). Given the high coverage and the lower length expected for a human transcriptome, the percentage of duplicated reads was high, reaching 69±5.5%, but the proportion of overrepresented sequences was low (<2%) indicating that all RNA sequenced libraries were diverse. The report of STAR alignments showed that on average 96.8±1.2% of the reads mapped to the reference genome with 91±2.3% mapping to unique loci (**Figure 2B**). The 3.15±1.26% of unmapped reads correspond mainly to reads with a short-alignment length (3.05±1.24%) that might result from the trimming process (trim of adaptor or low-quality bases, **Figure 2B**). Finally, as expected most of the MESOMICS reads mapped to mRNA structures including CDS and UTR regions (86.2±3.1%, **Figure 2C**).

DNA methylation data

DNA methylation analyses were performed in-house for 135 MPM samples from 122 patients, and an additional two technical replicates and three adjacent normal tissues, with Infinium EPIC DNA methylation beadchip platform (Illumina) which interrogates over 850,000 CpG sites, as described in [10]. Resulting raw IDAT files were processed using our in-house workflow (https://github.com/IARCbioinfo/Methylation_analysis_scripts, commit SHA bcfe876) in the R statistical programing environment using R packages minfi (v1.34.0; RRID:SCR_012830) and ENmix (v1.25.1), and consisted of the following four steps: pre-processing quality control, functional normalization, probe filtering, and finally beta and M-value computation.

During quality control checks on the raw data, one poor quality sample was identified when comparing per sample log2 methylated and unmethylated chip-wise median signal intensity (function getQC, minfi, **Figure 3A**) which was subsequently removed, and all samples displayed an overall *p*-detection value < 0.01 (function detectionP, minfi). Functional normalization, probe filtering, and beta and M-value computation were performed as described in [10]. The resulting dataset consisted of beta and M-values for 139 samples across 781,245 probes, with the M-value table containing nine -∞ values which were replaced by the next-lowest M-value for statistical analysis. The effect of normalization and probe-removal on DNA methylation profile is shown as beta density plots (pre-normalization in **Figure 3B** and post-normalization and probe removal in **Figure 3C**). Principal components analysis (PCA) was performed to detect batch effects, and to examine the effect of normalization, this was performed on a reduced number of samples (*n*=122, one tumor per patient, excluding technical replicates and normal tissues). Two datasets were used: (i) pre-normalized, unfiltered M-values (obtained from the GenomicRatioSet, function getM, minfi), and (ii) normalized and filtered M-values. Dataset (i) contained 2,478 CpGs with at least one NA value which were omitted before PCA, and 21,969 -∞ values were replaced with the next lowest M-value in the dataset, leaving an M-value matrix of 863,381 probes.

R package ade4 (v1.7-15) was used to calculate the first 10 principal components (function dudi.pca) across each dataset individually. We checked the association of the first 10 PCs with technical (chip, position on the chip, batch, sample well, sample provider, macrodissection), clinical (sex, age class, and smoking status), morphological (histopathological type, subtype, tumor percentage, necrosis and vessel level), and epidemiological variables (asbestos exposure, exposure probability, exposure frequency, and exposure intensity) using PC regression analysis, fitting separate linear models to each principal component with each of the 18 covariables of interest and adjusted the *p*-values for multiple testing (**Figures 3D** and **3E**). The first ten principal components in the normalized, filtered methylation data were significantly associated with type (PCs 1, 2, and 5), and sentrix chip position (PC 5, PC 8). The contribution of variance in the data from technical features before normalization was more pronounced, with sentrix chip and plate also being significant (PC 7), indicating functional normalization reduced technical batch effects on DNA methylation profile while retaining biological effects such as histological type. Before normalization, sex was significantly associated with PCs 2, 3, and 5, but not associated with any PCs in the normalized, filtered dataset. As probes on the sex chromosomes were removed after normalization, it was expected that this would reduce the effect of sex on variance in the dataset.

WGS variant calling in tumor-only samples

Copy number variants

Somatic Copy Number Alterations (SCNA) were called using our nextflow workflow purple-nf (https://github.com/IARCbioinfo/purple-nf, v1.0) that implements the PURPLE [23,24] software for matched and tumor-only WGS samples. To assess the quality of tumor-only PURPLE calls, a total of 57 matched-pairs were used as an evaluation set. Briefly, we ran PURPLE twice for each matched sample (**Figure 4A**): first using as input the matched-pairs, and second using only the tumor WGS as input. Subsequently we performed a direct comparison of the PURPLE tumor-only calls with their corresponding matched-pair calls for the following features: tumor purity, ploidy, number of segments, percentage of diploid, amplified, and deleted genome regions, as well as major and minor copy number states at the gene-level (**Figure 4**).

This benchmarking revealed a high concordance across all the evaluated metrics between tumor-only and matched PURPLE calls. Indeed, the agreement for purity (**Figure 4B**, $R = 0.988$), ploidy (**Figure 4C**, R=1), number of copy number segments per tumor (**Figure 4D**, $R = 0.981$), and percentage of genome changed (diploid, amplified, and deleted) exceeded a 0.98 correlation (**Figure 4 E-F**). Moreover, a high concordance was also observed at the gene level with major and minor copy number alleles reaching $R > 0.94$ (**Figure 4 G-H**). Finally, the only detected issue of tumor-only calls was observed near telomeric and centromeric regions, where artefactual focal peaks were detected (**Supplementary Figure 1**). These problematic regions were manually curated and the copy number segments overlapping such regions were removed from the tumor-only calls (see list of excluded segments in Table S1). In addition, because PURPLE does not round copy number values to 0, but rather penalizes negative values in the model fit, for all samples (both matched and tumor-only), following similar discussions with the PURPLE developers on the handling of negative values (https://github.com/hartwigmedical/hmftools/issues/102), we rounded slightly negative copy number estimates (in]-0.5,0[) to 0 and excluded largely negative copy number estimates (<-0.5) from subsequent analyses, because they suggest high noise in the read depth and are thus unreliable calls. Note that in total (including segments with largely negative values), we excluded only 0.26% of the total segment length.

Calling somatic point mutations and structural variants.

Unlike copy number variants whereby the software (PURPLE) directly generated highly accurate results in tumor-only mode without any post-processing, for point mutations and structural variants (SVs) direct outputs from calling pipelines and typical filters (i.e. removing variants matching germline databases) did not remove at high accuracy the germline variants present in tumor-only MPM WGS. Therefore, we trained and evaluated the performance of a supervised machine learning model based on a random forest (RF [25]) for distinguishing germline from somatic variants in tumor-only WGS (**Figure 5A, Supplementary Note 1**).

Point mutations were called using Mutect2 (RRID:SCR_000559) using our nextflow pipeline Mutect2-nf (https://github.com/IARCbioinfo/mutect-nf, v2.2b). The matched samples were used as input for training and evaluating the performance of the random forest (RF) model (**Figure 5A**) for classifying germline and somatic mutations. The random forest model for point mutations includes a total of 20 features divided into three main classes, namely: associated with external databases (gnomAD r3.0[26] RRID:SCR_014964 and COSMIC v90 [26] RRID:SCR_002260), genomic location/impact/signatures [27], and features obtained directly from the point mutation variant caller – Mutect2 (**Figure 5B, Supplementary Note 1**). The matching of variants against reference databases was performed using bcftools (v1.10.2, annotate function RRID:SCR_005227)[28]. For training the RF model a total of 46 tumors with matched normal MPM whole-genome sequences called with both the tumor-only and matched modes of Mutect2 [23] (RRID:SCR_000559) were used (**Figure 5A**). The matched somatic calls (ground-truth) were used to annotate the variants of the tumor-only WGS into germline and somatic classes.

The training and evaluation of models were performed using 75% and the remaining 25% of the dataset, respectively. A grid search revealed that the optimum parameters were mtry=8, ntree=1000, and nodesize=5, reaching a model accuracy of 0.9276 in the testing set. A random forest model for SNVs (rfvs01) was trained with the optimum parameters using a total of 326,388 (80%) variants (1:1 ratio). Analysis of the feature importance revealed that the allele frequency (AF) is the most discriminative feature included in the model (**Figure 5B**). For indels, a random forest model (rfvi01) was built with the same optimal parameters using a total of 337,442 variants (1:1 ratio, including 305,988 SNVs and 31,454 indels) and removing the SNVs feature. The performance of the optimal RF-models for SNVs and indels reached an accuracy of 0.926 and 0.924, respectively (**Figure 5C**). The trained RF models (rfvs01 and rfvi01) were used to classify a total of 1,454,942 variants (SNVs=1,317,200 and indels=137,742) of which 217,436 variants (including SNVs and indels) were classified as somatic.

Large genomic rearrangements were detected using a consensus variant calling approach including SvABA (v1.1.0) [29], Manta (v1.6.0) [30], and Delly (v0.8.3, RRID:SCR_004603) [31] followed by subsequent integration with SURVIVOR (v1.0.7) [32]. Our nextflow pipeline implementing the consensus variant calling approach for matched WGS is available at https://github.com/IARCbioinfo/sv_somatic_cns.

Like for point mutations, we implemented custom random forest models to distinguish at high accuracy somatic from germline SVs in tumor-only MPM samples (**Figure 5A, Supplementary Note 1**). The RF models were composed of a total of 19 features based on external databases (gnomAD [26] RRID:SCR_014964 and PCAWG [33]), a custom panel of normal, genomic regions (Cosmic v90 RRID:SCR_002260, Gencode v33 RRID:SCR_014966 and

PhastCons[34] RRID:SCR_003204), and SV features obtained directly from each SV caller (**Figure 5D**). The training (75%) and evaluation (25%) of the random forest model for each SV caller were performed using a total of 12,454, 16,720, and 12,264 SVs at 1:1 somatic: germline proportions for Delly [29] (RRID:SCR_004603), Manta [30], and SvABA [31], respectively. All three SV random forest models were trained using the default random forest parameters (mtry=4, ntree=400 and nodesize=1). The precision, recall, and accuracy achieved by each model were 0.905±0.009, 0.87±0.016, and 0.889±0.010, respectively (**Figure 5E**). The most important features of the models were the number of PON SVs around both breakpoints, SV alternative allele frequency, SV read depth, and SV length (**Figure 5D**). We performed additional comparisons by SV type, SV length, and number of SVs as a function of the purity of samples, WGS type (matched tumor-normal, tumor-only) and MPM subtype and did not observe any significant difference between SVs called in the tumor-only or matched WGS MESOMICS series (**Figure 5F** and **5G**). The SV calls for the MESOMICS tumor-only samples include a total of n=8,229 SVs, which combined with the SVs called in the matched series gave a total of n=12,914 (**Figure 5H**).

Our results demonstrate that our methodology is highly accurate and robust to call point mutations and structural variants in tumor-only WGS datasets for which a series of matched tumor-normal samples are available. The source code and the random forest models implemented for MPM are available in the Github repositories https://github.com/IARCbioinfo/RF-mut-f and https://github.com/IARCbioinfo/ssvht for point mutations and structural variants, respectively.

Data Validation

Muti-omic sample matching

The software NGSCheckMate [35] was used to check the match between sequencing modalities of a given MESOMICS patient. NGSCheckMate was run using our nextflow implementation available at https://github.com/IARCbioinfo/NGSCheckMate-nf (v1.1a). NGSCheckMate, using WGS and RNA-seq, confirmed that the majority of MESOMICS samples were correctly paired (**Figure 6A**, black segments). However, NGSCheckMate discovered that the WGS of MESO_094_T and MESO_096_T matched (**Figure 6A**, red segments). Further examination of these samples confirmed that both WGS come from the same patient but were annotated differently during sample collection. In addition, the RNA-seq replicate named MESO_054_TR1 matched with the group of samples coming from patient MESO_051. After sequencing a second RNA-seq aliquot from MESO_054_T, named MESO_054_TR2, and re-performing the NGSCheckMate analysis, we confirmed a miss-annotation of these RNA-seq samples and proceeded to rename them as MESO_051_TR1 and MESO_051_TR2, respectively. After the aforementioned corrections, all the

sequencing modalities at the sample and patient level were correctly paired for the complete MESOMICS cohort.

Sex validation

We registered the sex (M for male or F for female) data for all the 124 patients of the MESOMICS cohort. We validated the sex annotation based on the concordance of whole-genome, transcriptome, and methylome data (**Figure 6B-D**). First, the concordance between sex reported in the clinical data and WGS data was assessed by computing the total coverage on X and Y chromosomes (**Figure 6B**). Interestingly, some tumors from male individuals displayed an intermediate coverage on chromosome Y between other male and female cases, compatible with the large copy losses identified in our study like for example the tumor from MESO_071. Second, the concordance between sex reported in the clinical data and sex chromosome gene expression patterns (transcriptome) was performed by comparing the sum of variance-stabilized read counts (vst function from R package DESeq2, v.1.14.1 RRID:SCR_015687) of each sample on the X and Y chromosomes (**Figure 6C**). Third, the concordance between the sex reported in the clinical data and the methylation data was assessed using a predictor based on the median total intensity on sex-chromosomes, with a cut-off of -2 log2 estimated copy number (function getSex from minfi, v.1.34.0 RRID:SCR_012830, **Figure 6D**). The only sex discordance was observed in MESO_071 tumor sample due to somatic copy number losses in the Y chromosome, but the whole genome sequencing from matched blood confirmed that this patient was male (Figure 6B). In summary, the sex data of the MESOMICS cohort was validated using a multi-omic approach that confirmed the sex of all the MESOMICS samples.

Purity

Tumor purity has been estimated from three independent data sources: from genomic data using PURPLE, from transcriptomic data using quanTIseq [36], and through pathological review. We performed pairwise comparisons between these three estimates and found significant correlations between pathological and each molecular estimate (q-value = 8.40×10^{-3} and 2.49×10^{-4} with transcriptomic and genomic purity, respectively). The transcriptomic and genomic estimates are significantly correlated as well (q-value = 4.05×10^{-4}, **Supplementary Figure 2**). Of note, four samples (MESO_050_T, MESO_058_T2, MESO_059_T1, and MESO_076_T) have been excluded from the analyses of genomic estimates of purity because no somatic CNVs were identified and thus purity could not be estimated by PURPLE . The four samples all had low pathological estimates ([0.1-0.4]) and moderate transcriptomic estimates of purity ([0.53-0.69]).

An integrative and interactive MPM phenotypic map

Task specialization analysis using Pareto

In order to integrate the MESOMICS multi-omic data and investigate the association between the detected genomic events in this new large genomic cohort and the observed MPM phenotypes, we firstly performed a multi-omic summary of MPM using MOFA [37] and secondly performed a task specialization analysis to identify MPMs with natural selection for specific cancer tasks (see [10]). We performed task specialization analyses using the well-established Pareto optimum theory (ParetoTI method) [11]. The Pareto front model has been fitted to different sets of samples using the ParetoTI R package (https://github.com/vitkl/ParetoTI, v0.1.13) on MOFA latent factors (LFs), restricted to LF1, LF2, LF3, and LF4 due to their association with survival and extreme phenotypes (see [10]). In brief, according to the theory a molecular map would take a particular shape (polyhedra) if a trade-off exists between several cancer tasks performed by the tumors. Using MOFA axes, we found a triangle (polyhedra with three vertices) corresponding to *k* = 3 archetypes in the LF2-LF3 space. According to the Pareto optimum theory, this pattern results from natural selection for cancer tasks, with specialized tumors close to the vertices of the triangle (representing archetypes), and generalists in the center. We have also replicated the same analyses (MOFA and ParetoTI) on the previously published multi-omic studies from Bueno *et al.* [2] (*n=181* fresh-frozen surgically resected primary tissue) and Hmeljak *et al.* [3] (*n=73* fresh-frozen surgically-resected or biopsy tissue). R scripts to prepare matrices for each omic layer, as well as scripts to run MOFA and the Pareto analysis for the three cohorts are available in the Github repository dedicated for this data note paper at https://github.com/IARCbioinfo/MESOMICS_data.

Biological interpretation of the MPM phenotypic map

We inferred each archetype's phenotype by performing integrative gene set enrichment analysis on the expression data and identified the following cancer tasks and tumor phenotypes: Cell division, Tumor-immune-interaction, and Acinar phenotype (see [10]). Tumors specialized in the Cell division task displayed upregulation of pathways within the "cell division" task as reported by Hausser et al. [38] in multiple tumor types. This phenotype was enriched for non-epithelioid tumors and presented higher levels of necrosis, higher grade, high expression of hypoxia response pathways, and greater percentage of infiltrating neutrophils that are innate immune response cells. Cell division specialization was supported by the high expression levels of the proliferation marker *MKI67*, and increased genomic instability. Tumors specialized in the Tumor-immune-interaction task carried upregulated immune-related pathways, high expression of immune checkpoint genes, and high immune infiltration with an enrichment for adaptive-response cells: lymphocytes B, T-CD8+, and T-reg. The last extreme phenotype was characterized by samples with Acinar morphology, presenting a very structured tissue organization with

epithelial cells tightly linked into tubular structures, and correlated with the presence of monocytes and NK cells (innate immune response cells). This phenotype presented the lowest epithelial-mesenchymal transition (EMT) score [39], with overexpression of epithelial markers such as cell-adhesion molecules, corroborating the importance of tissue organization in this phenotype, and also low levels of *MKI67* expression, indicating slow growth. Altogether these data provide a biological understanding for the molecular and phenotypic heterogeneity characteristic of MPM tumors.

Reuse potential

The MESOMICS project represents the most comprehensive molecular characterization of MPM to date, made possible by inclusion of the largest WGS dataset yet reported, and by the depth of the analyses undertaken. Multi-omics integration and biological interpretation through the lense of Pareto theory has allowed us to uncover three specialized MPM tumor profiles [10]. In order to replicate these findings while minimizing batch effects associated with bioinformatics data processing, we have accessed and reprocessed the raw data from previously published MPM multi-omics studies [2,3] using the same analytical procedures. A by-product of this laborious work is the creation of the largest (*n=374* samples in total) existing harmonized dataset of MPM multi-omics data.

In order to maximize the reuse potential of this dataset, we have also harmonized the available clinical, epidemiological and morphological data from these three cohorts. In addition to providing the raw data, the full list of genomic variants and the entire matrices of expression and methylation levels, we provide a curated and harmonized list of molecular features (e.g. immune cell composition, measures of genomic instability, presence of whole genome duplication, copy number in recurrently altered regions, driver genes mutational status, expression level of some relevant genes etc.) across all samples (**Supplementary Table S2**).

This MPM phenotypic map has been shared on the TumorMap web portal [12], offering an interactive visualization of this data in the tumor phenotypes space (Cell division, Tumor-immune-interaction, and Acinar phenotype), including all the harmonized clinical, morphological, epidemiological, and molecular data attributes mentioned above. The TumorMap interface provides an interactive way to explore and navigate through the map, where each sample is represented by a dot localized according to its position in the phenotype space (**Figure 7**). The attributes can be used to change colors and filter samples, perform statistical tests, and new attributes can be derived from pre-existing ones using set operations. This flexible and user-friendly interface will enable new hypotheses to be tested without computational expertise, and expands the reuse potential of the dataset. The TumorMap is available at https://tumormap.ucsc.edu/RCG_MESOMICS/MPM_Archetypes.

Conclusion

We demonstrated that we provide a high-quality multi-omic dataset of malignant pleural mesothelioma, including the largest whole-genome sequencing dataset of malignant pleural mesothelioma to date, consisting of both raw and processed data, and important molecular phenotypes. By homogenizing the clinical, epidemiological, morphological and molecular data of our new series with the two previously published MPM multi-omics data series, we have created an unprecedented dataset for this rare cancer in terms of both size and detail. We provide all the resources to reproduce our analyses, as well as a user-friendly interactive visualization tool, which will contribute to advancing biological knowledge of this deadly disease. As most patients with MPM will survive to 2nd or 3rd line systemic therapy, future studies will be needed to describe the molecular landscape of MPM at these time points to develop effective precision medicine strategies.

Availability of Supporting Data and Materials

The data used in this manuscript are available in the European Genome-phenome Archive (EGA), which is hosted at the EBI and the Centre for Genomic Regulation (CRG), under the accession number EGAS00001004812 (https://ega-archive.org/studies/EGAS00001004812); download requires approval from the data access committee EGAC00001001811 (https://ega-archive.org/dacs/EGAC00001001811 ; email Dr Matthieu Foll at follm@iarc.fr), and then installing the EGA download python client and its dependencies (python3 and pip3; see instructions at https://github.com/EGA-archive/ega-download-client and a video tutorial at https://embl-ebi.cloud.panopto.eu/Panopto/Pages/Viewer.aspx?id=be79bb93-1737-4f95-b80f-ab4300aa6f5a). R scripts are available in the github repository https://github.com/IARCbioinfo/MESOMICS_data. The interactive molecular phenotypic map is available at https://tumormap.ucsc.edu/RCG_MESOMICS/MPM_Archetypes.

Figure legends

Figure 1. Quality control of Whole-Genome Sequencing (WGS) data. A) Number of reads per WGS type. B) Mean sequence quality score as a function of the position in the read in base pairs. Green lines correspond to files that passed the most stringent QC filters of software FastQC; orange lines correspond to files that passed a less stringent filter; and red to files that did not pass the filters. C) Percentage of aligned reads to the reference human genome. D) Cumulative genome fraction computed directly from the BAM files.

Figure 2. Quality control of RNA Sequencing (RNA-seq) data. A) Distribution of sequence quality scores in Phred scale for 2x75bp and 2x100bp read pairs. B) STAR alignment scores. C) Distribution of reads mapped to different genomic regions.

Figure 3. Quality control of EPIC array sequencing data. A) Signal intensity plot. Log2 methylated and unmethylated median signal intensity plot of 140 samples. One sample (coloured red) fell below the cut-off of 10.5 and was subsequently removed from analysis. B) Pre-normalisation beta density plot. Beta density plot of 140 samples across 865,859 probes, coloured by tumour/normal type, prior to functional normalisation. C) Post-normalisation and filtering beta density plot. Beta density plot of 139 samples across 781,245 probes, coloured by tumour/normal type, following functional normalisation and removal of cross-reactive, sex-chromosome, SNP, and failed (p-detection > 0.01) probes. D) Association of technical and clinical variables with pre-normalisation principal components. Association of technical and clinical variables with principal components one to ten, for 122 samples. Principal components calculated from M-values of 863,381 pre-normalised probes. E) Association of technical and clinical variables with post-normalisation principal components. Principal components calculated from M-values of 781,245 probes following functional normalization and probe removal.

Figure 4. Performance of somatic copy number variant calling from tumor-only samples. A) Schematic of the benchmarking procedure. Comparison of Tumor/Normal and Tumor-only calling for B) Purity, C) Ploidy, D) Number of copy number segments, E) Diploid proportion, F) Percentage of deleted genome, G) Major allele copy number and H) Minor allele copy number.

Figure 5. Performance of somatic point mutation and structural variant calling from tumor-only samples. A) Schematic of the benchmarking procedure. B) Random forest model features and their ranking for predicting somatic SNV and Indels. C) Performance metrics (precision, recall, accuracy) for classifying somatic point mutations with the best performing RF models. D) SVs Random Forest model features and their ranking for predicting somatic SVs. E) Performance

metrics for classifying somatic SVs. F) Number of SVs as function of WGS type. Mean comparison between WGS types was performed using a t-test with no significant (ns) result found. G) Number of SVs as function of tumor purity. A linear model (`number_sv ~Purity*WGS_type*SubType`) was built to predict the number of SVs, no significant coefficients (p.value < 0.05) were found. H) Venn diagram of the final consensus MESOMIC SVs set.

Figure 6. Applications of data validation using multi-omics data A) Network of matching WGS and RNA-seq samples, as computed by software NGSCheckmate. Edge transparency corresponds to the Pearson Correlation r between SNP panel allelic fractions; node color and surrounding color correspond respectively to the techniques (WGS or RNA-seq) and to the tissue type (Normal, Matched samples or T-only samples). B-D Sex reclassification and multi-omic validation of reported clinical sex. B) Total exome reads coverage on the X and Y chromosomes for each sample. C) Total expression level of each sample on the X and Y chromosomes (in variance-stabilized read counts). D) Median methylation array total intensity on the X and Y chromosomes. In panel (B), point colors correspond to the WGS groups: normal samples in light green, tumor samples with matched normals (Match) in dark green, and tumor samples without matched normal (T-only) in red. In each panel, filled polygons correspond to the sexes given by the clinical annotations (blue for male, red for female). In panel D) point colors correspond to the sexes predicted by the DNA methylation QC. Samples with discordant reported clinical sex and molecular patterns on sex chromosomes are indicated.

Figure 7. MPM molecular phenotypic map. Screen capture from the TumorMap portal, using the hexagonal grid view, each point representing a MPM sample in the triangular phenotypic space: cell division (left vertice), tumor-immune-interaction (top vertice), and acinar phenotype (right vertice). Point colors correspond to the histological types and can be interactively changed by the users on the web portal.

Supplemental files

Supplementary Figure S1. MPM CNV cohort profile aCNViewer plot [40] from Tumor-matched called as Tumor-Only (top), Tumor-only (middle), and Tumor-only after filtering (bottom). The circled regions correspond to artifactual peaks when calling CNVs with the Tumor-only mode of PURPLE. The aforementioned genomic regions were identified, filtered and are provided in Supplementary Table S1.

Supplementary Figure S2. Correlation between purity estimates from three different omic purity measurements: the proportion of DNA material from the tumor (genomic estimate of purity), the complement proportion of infiltrating immune cells (transcriptomic estimate of purity), and the amount of tumor tissue in the observed slide (pathological estimate of purity). (A) between transcriptomic and pathological estimates, (B) between genomic and pathological estimates, and (C) between genomic and transcriptomic estimates. In these three panels, q-values and coefficient r correspond to Pearson's correlation tests.

Supplementary Table S1. List of excluded genomic regions identified as artifactual when calling CNVs using PURPLE Tumor-only mode.

Supplementary Table S2. Harmonized and curated molecular, clinical, epidemiological and morphological data from our MESOMICS cohort, and the two previously published MPM multi-omics data [2,3]. This table can be explored interactively on the UCSC TumorMap web portal.

Supplementary Note 1. Additional details of the point mutation and structural variant calling for tumor-only MPM WGS samples.

List of abbreviations

ABRA: Assembly-Based Realigner; BAM: Binary Alignment Map; CDS: coding sequence; CNV: Copy Number Variant; CpG: cytosine–phosphate–guanine; EGA: European Genome-phenome Archive; EMBL-EBI: European Bioinformatics Institute; GATK: Genome Analysis Toolkit; IARC: International Agency for Research on Cancer; MME: malignant mesothelioma epithelioid; MMB: malignant mesothelioma biphasic; MMS: malignant mesothelioma sarcomatoid; MPM: malignant pleural mesothelioma; PCA: principal component analysis; QC: quality control; FR: Random Forest; RNA-Seq: RNA sequencing; SNP: single-nucleotide polymorphism; STAR: Spliced Transcripts Alignment to a Reference; SV: Structural Variant; TES: transcription end site; TSS: transcription start site; UCSC: University of California Santa Cruz; vst: variance-stabilized transformation; WGS: whole-genome sequencing.

Ethics approval

These data belong to the MESOMICS project, which has been approved by the IARC Ethical Committee.

Competing interests

Where authors are identified as personnel of the International Agency for Research on Cancer/World Health Organisation, the authors alone are responsible for the views expressed in this article and they do not necessarily represent the decisions, policy or views of the International Agency for Research on Cancer/World Health Organisation.

Funding

This work has been funded by the French National Cancer Institute (INCa, PRT-K 2016-039 to L.F.C. and M.F.), the Ligue Nationale contre le Cancer (LNCC 2017 and 2020 to L.F.C. and M.F.). L.M. has a fellowship from the LNCC.

Author’s contributions

Conceptualization, M.F., N.A., L.F.-C; Methodology, M.F., N.A., L.F.-C., L.M., A.DG., A.S.-O.; Software, A.DG., L.M., A.S.-O, C.V., N.A.; Validation, A.DG., L.M., A.S.-O, N.A.; Formal Analyses, A.DG., L.M., A.S.-O, C.V., N.A.; Investigation, A.DG., L.M., A.S.-O, C.V., N.A.; Data Curation, A.DG., L.M., A.S.-O, C.V., N.A.; Writing – Original Draft, A.DG., M.F., L.M., N.A., A.S.-O.; Writing – Review & Editing, A.DG., M.F., L.M., N.A., A.S.-O., L.F.-C.; Visualization, A.DG, L.M., N.A., A.S.-O., C.V.; Supervision, L.F.-C., M.F., N.A.; Project Administration, L.F.-C., M.F., L.M., N.A.; Funding Acquisition, L.F.-C., M.F., N.A.

Acknowledgements

This study is part of the MESOMICS project within the Rare Cancers Genomics initiative (www.rarecancersgenomics.com). We also acknowledge the Cologne Centre for Genomics (Cologne, Germany) and the Centre National de Recherche en Génomique Humaine (Evry, France) for generating high-quality sequencing data, as well as the Epigenomics and Mechanisms branch at IARC for generating high-quality methylation data. The results published here are part based upon data generated by the TCGA Research Network: https://www.cancer.gov/tcga.

References

1. WHO Classification of Tumours Editorial Board. Thoracic Tumours. International Agency for Research on Cancer.;

2. Bueno R, Stawiski EW, Goldstein LD, Durinck S, De Rienzo A, Modrusan Z, et al.. Comprehensive genomic analysis of malignant pleural mesothelioma identifies recurrent mutations, gene fusions and splicing alterations. *Nat Genet*. 48:407–162016;

3. Hmeljak J, Sanchez-Vega F, Hoadley KA, Shih J, Stewart C, Heiman D, et al.. Integrative Molecular Characterization of Malignant Pleural Mesothelioma. *Cancer Discov*. 8:1548–652018;

4. Alcala N, Mangiante L, Le-Stang N, Gustafson CE, Boyault S, Damiola F, et al.. Redefining malignant pleural mesothelioma types as a continuum uncovers immune-vascular interactions. *EBioMedicine*. 48:191–2022019;

5. Blum Y, Meiller C, Quetel L, Elarouci N, Ayadi M, Tashtanbaeva D, et al.. Dissecting heterogeneity in malignant pleural mesothelioma through histo-molecular gradients for clinical applications. *Nat Commun*. 10:13332019;

6. Fernandez-Cuesta L, Mangiante L, Alcala N, Foll M. Challenges in lung and thoracic pathology: molecular advances in the classification of pleural mesotheliomas. *Virchows Arch*. 478:73–802021;

7. Cortés-Ciriano I, Lee JJ-K, Xi R, Jain D, Jung YL, Yang L, et al.. Comprehensive analysis of chromothripsis in 2,658 human cancers using whole-genome sequencing. *Nat Genet*. 52:331–412020;

8. Kim H, Nguyen N-P, Turner K, Wu S, Gujar AD, Luebeck J, et al.. Extrachromosomal DNA is associated with oncogene amplification and poor outcome across multiple cancers. *Nat Genet*. 52:891–72020;

9. Quinton RJ, DiDomizio A, Vittoria MA, Kotýnková K, Ticas CJ, Patel S, et al.. Whole-genome doubling confers unique genetic vulnerabilities on tumour cells. *Nature*. 590:492–72021;

10. Mangiante L, Alcala N, Di Genova A, Sexton-Oates A. Disentangling heterogeneity of Malignant Pleural Mesothelioma through deep integrative omics analyses. *bioRxiv*. biorxiv.org; 2021;

11. Hausser J, Alon U. Tumour heterogeneity and the evolutionary trade-offs of cancer. *Nat Rev Cancer*. 20:247–572020;

12. Newton Y, Novak AM, Swatloski T, McColl DC, Chopra S, Graim K, et al.. TumorMap: Exploring the Molecular Similarities of Cancer Samples in an Interactive Portal. *Cancer Res*. 77:e111–42017;

13. : IARCbioinfo/MESOMICS_data GitHub repository. https://github.com/IARCbioinfo/MESOMICS_data Accessed 2022 Jul 6.

14. Zalcman G, Mazieres J, Margery J, Greillier L, Audigier-Valette C, Moro-Sibilot D, et al.. Bevacizumab for newly diagnosed pleural mesothelioma in the Mesothelioma Avastin Cisplatin Pemetrexed Study (MAPS): a randomised, controlled, open-label, phase 3 trial. *Lancet*. 387:1405–142016;

15. Baas P, Scherpereel A, Nowak AK, Fujimoto N, Peters S, Tsao AS, et al.. First-line nivolumab

plus ipilimumab in unresectable malignant pleural mesothelioma (CheckMate 743): a multicentre, randomised, open-label, phase 3 trial. *Lancet*. 397:375–862021;

16. Di Tommaso P, Chatzou M, Floden EW, Barja PP, Palumbo E, Notredame C. Nextflow enables reproducible computational workflows. *Nat Biotechnol*. 35:316–92017;

17. Gabriel AAG, Mathian E, Mangiante L, Voegele C, Cahais V, Ghantous A, et al.. A molecular map of lung neuroendocrine neoplasms. *Gigascience*. 2020; doi: 10.1093/gigascience/giaa112.

18. Dobin A, Davis CA, Schlesinger F, Drenkow J, Zaleski C, Jha S, et al.. STAR: ultrafast universal RNA-seq aligner. *Bioinformatics*. 29:15–212013;

19. Mose LE, Perou CM, Parker JS. Improved indel detection in DNA and RNA via realignment with ABRA2. *Bioinformatics*. 35:2966–732019;

20. Van der Auwera GA, O'Connor BD. Genomics in the Cloud: Using Docker, GATK, and WDL in Terra. "O'Reilly Media, Inc.";

21. Wang L, Wang S, Li W. RSeQC: quality control of RNA-seq experiments. *Bioinformatics*. 28:2184–52012;

22. Ewels P, Magnusson M, Lundin S, Käller M. MultiQC: summarize analysis results for multiple tools and samples in a single report. *Bioinformatics*. 32:3047–82016;

23. Priestley P, Baber J, Lolkema MP, Steeghs N, de Bruijn E, Shale C, et al.. Pan-cancer whole-genome analyses of metastatic solid tumours. *Nature*. 575:210–62019;

24. Cameron DL, Baber J, Shale C, Papenfuss AT, Valle-Inclan JE, Besselink N, et al.. GRIDSS, PURPLE, LINX: Unscrambling the tumor genome via integrated analysis of structural variation and copy number. bioRxiv.

25. Liaw A, Wiener M, Others. Classification and regression by randomForest. *R news*. 2:18–222002;

26. Karczewski KJ, Francioli LC, Tiao G, Cummings BB, Alföldi J, Wang Q, et al.. The mutational constraint spectrum quantified from variation in 141,456 humans. *Nature*. 581:434–432020;

27. Alexandrov LB, Kim J, Haradhvala NJ, Huang MN, Tian Ng AW, Wu Y, et al.. The repertoire of mutational signatures in human cancer. *Nature*. 578:94–1012020;

28. Danecek P, Bonfield JK, Liddle J, Marshall J, Ohan V, Pollard MO, et al.. Twelve years of SAMtools and BCFtools. *Gigascience*. 2021; doi: 10.1093/gigascience/giab008.

29. Wala JA, Bandopadhayay P, Greenwald NF, O'Rourke R, Sharpe T, Stewart C, et al.. SvABA: genome-wide detection of structural variants and indels by local assembly. *Genome Res*. 28:581–912018;

30. Chen X, Schulz-Trieglaff O, Shaw R, Barnes B, Schlesinger F, Källberg M, et al.. Manta: rapid detection of structural variants and indels for germline and cancer sequencing applications. *Bioinformatics*. 32:1220–22016;

31. Rausch T, Zichner T, Schlattl A, Stütz AM, Benes V, Korbel JO. DELLY: structural variant discovery by integrated paired-end and split-read analysis. *Bioinformatics*. 28:i333–92012;

32. Jeffares DC, Jolly C, Hoti M, Speed D, Shaw L, Rallis C, et al.. Transient structural variations have strong effects on quantitative traits and reproductive isolation in fission yeast. *Nat*

Commun. 8:140612017;

33. Li Y, Roberts ND, Wala JA, Shapira O, Schumacher SE, Kumar K, et al.. Patterns of somatic structural variation in human cancer genomes. *Nature*. 578:112–212020;

34. Pollard KS, Hubisz MJ, Rosenbloom KR, Siepel A. Detection of nonneutral substitution rates on mammalian phylogenies. *Genome Res*. 20:110–212010;

35. Lee S, Lee S, Ouellette S, Park W-Y, Lee EA, Park PJ. NGSCheckMate: software for validating sample identity in next-generation sequencing studies within and across data types. *Nucleic Acids Res*. 45:e1032017;

36. Finotello F, Mayer C, Plattner C, Laschober G, Rieder D, Hackl H, et al.. Molecular and pharmacological modulators of the tumor immune contexture revealed by deconvolution of RNA-seq data. *Genome Med*. 11:342019;

37. Argelaguet R, Velten B, Arnol D, Dietrich S, Zenz T, Marioni JC, et al.. Multi-Omics Factor Analysis-a framework for unsupervised integration of multi-omics data sets. *Mol Syst Biol*. 14:e81242018;

38. Hausser J, Szekely P, Bar N, Zimmer A, Sheftel H, Caldas C, et al.. Tumor diversity and the trade-off between universal cancer tasks. *Nat Commun*. Nature Publishing Group; 10:1–132019;

39. Mak MP, Tong P, Diao L, Cardnell RJ, Gibbons DL, William WN, et al.. A Patient-Derived, Pan-Cancer EMT Signature Identifies Global Molecular Alterations and Immune Target Enrichment Following Epithelial-to-Mesenchymal Transition. *Clin Cancer Res*. 22:609–202016;

40. Renault V, Tost J, Pichon F, Wang-Renault S-F, Letouzé E, Imbeaud S, et al.. aCNViewer: Comprehensive genome-wide visualization of absolute copy number and copy neutral variations. *PLoS One*. 12:e01893342017;

Figure 1
Click here to access/download;Figure;file-1.pdf

Figure 1

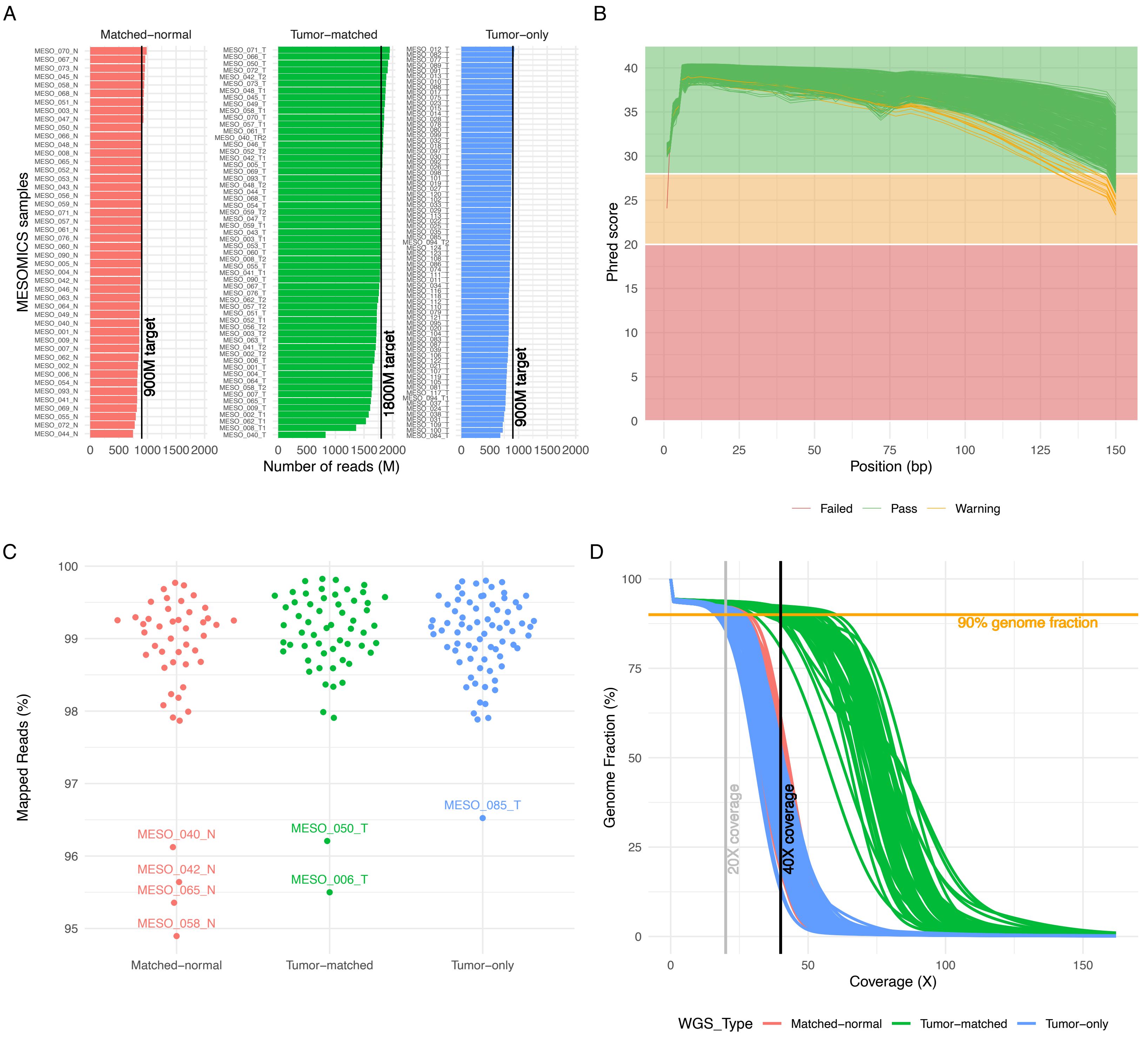

Figure 2

Click here to access/download;Figure;Figure2.pdf

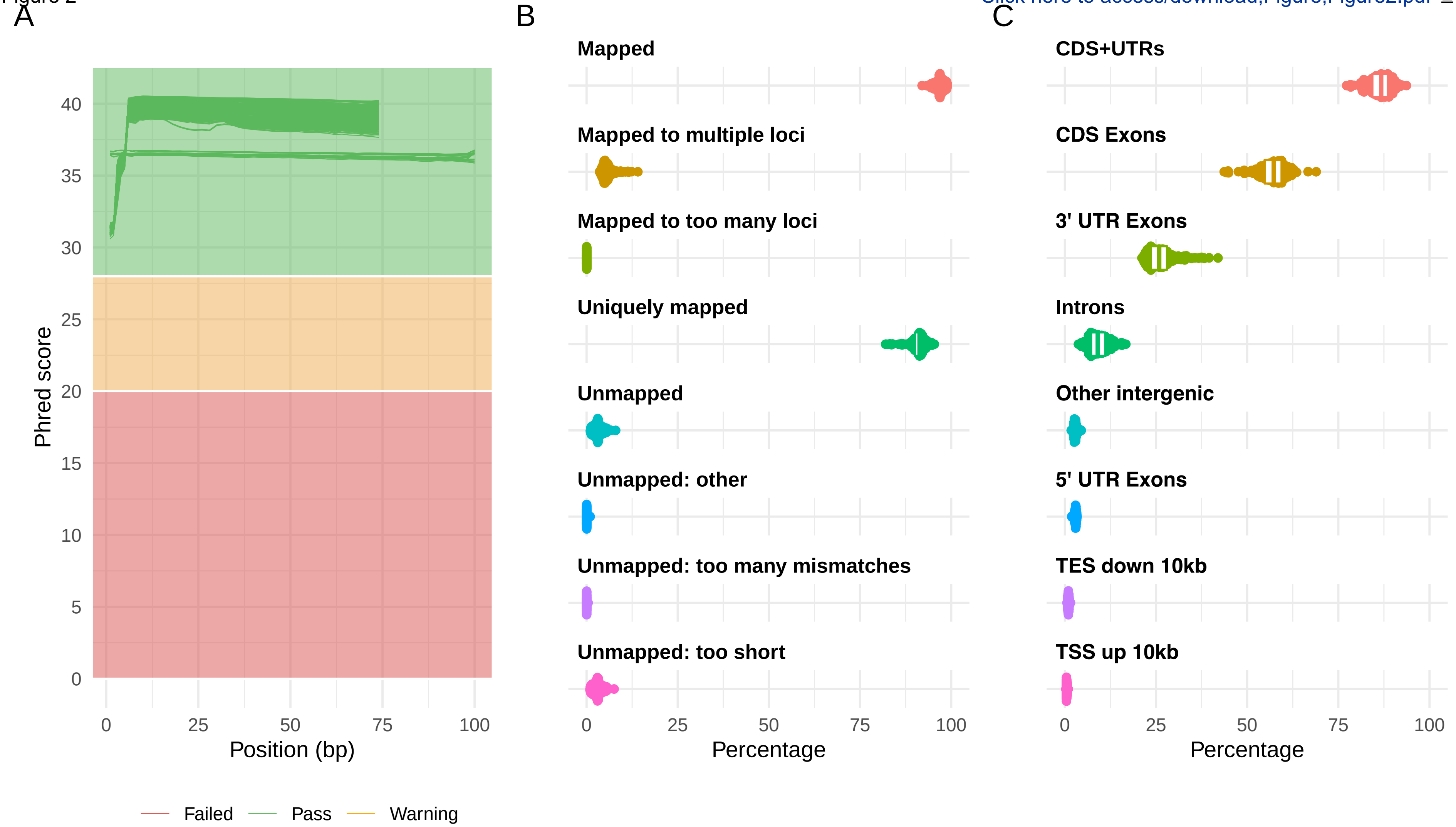

Figure 3
Click here to access/download;Figure;file-3.pdf

Figure 3

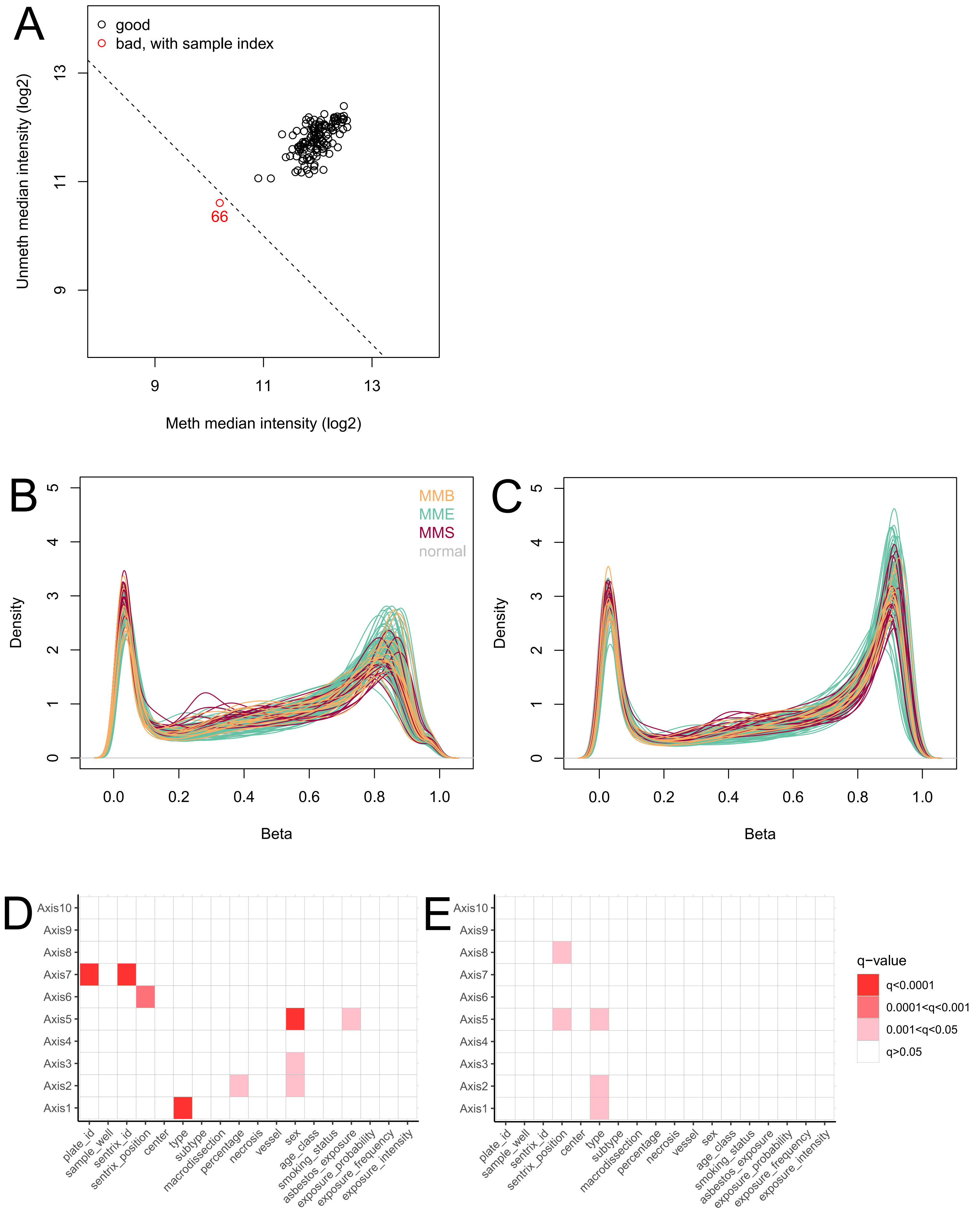

Click here to access/download;Figure;file-4.pdf

Figure 4

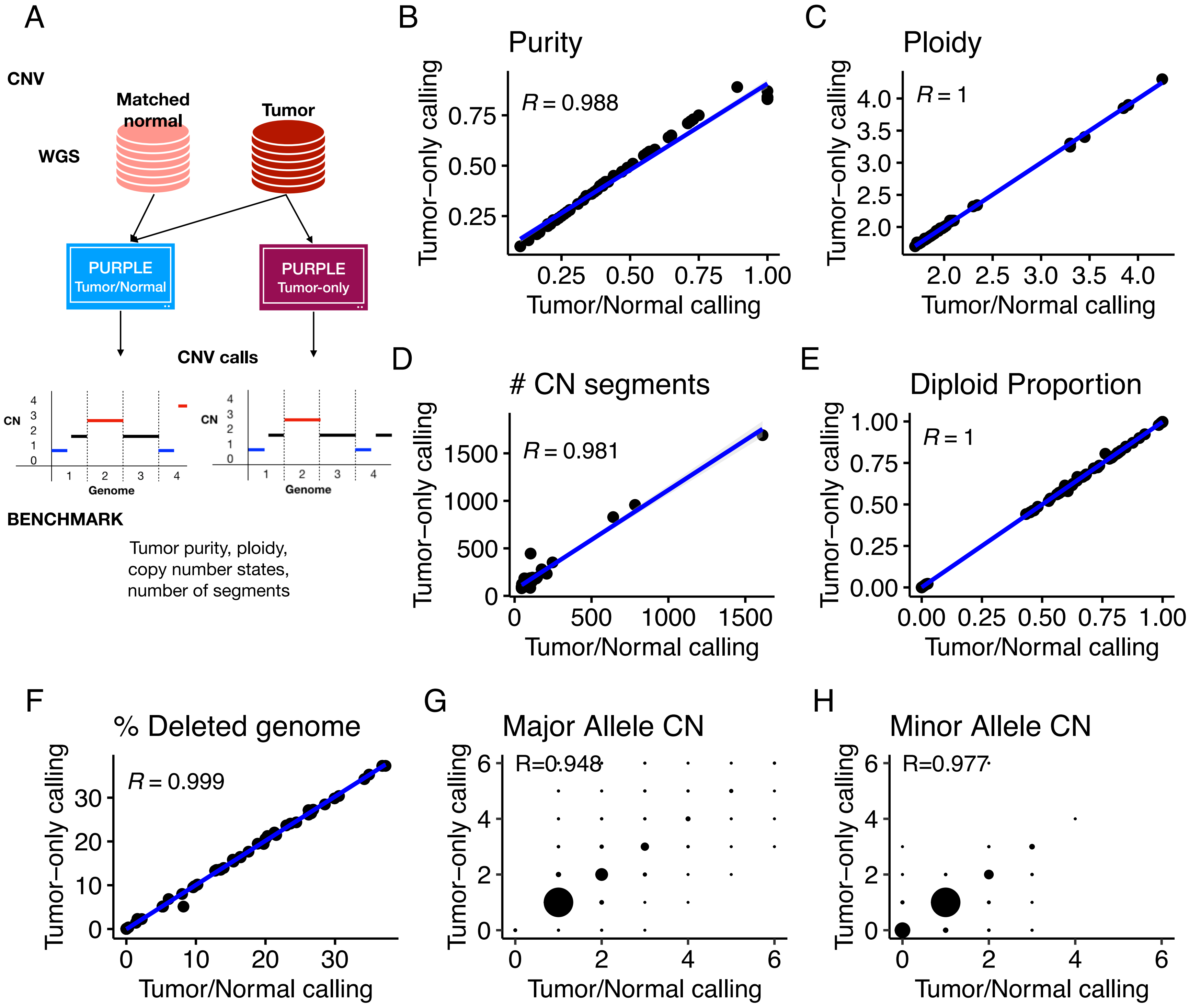

Figure 5

Click here to access/download;Figure;file-5.pdf

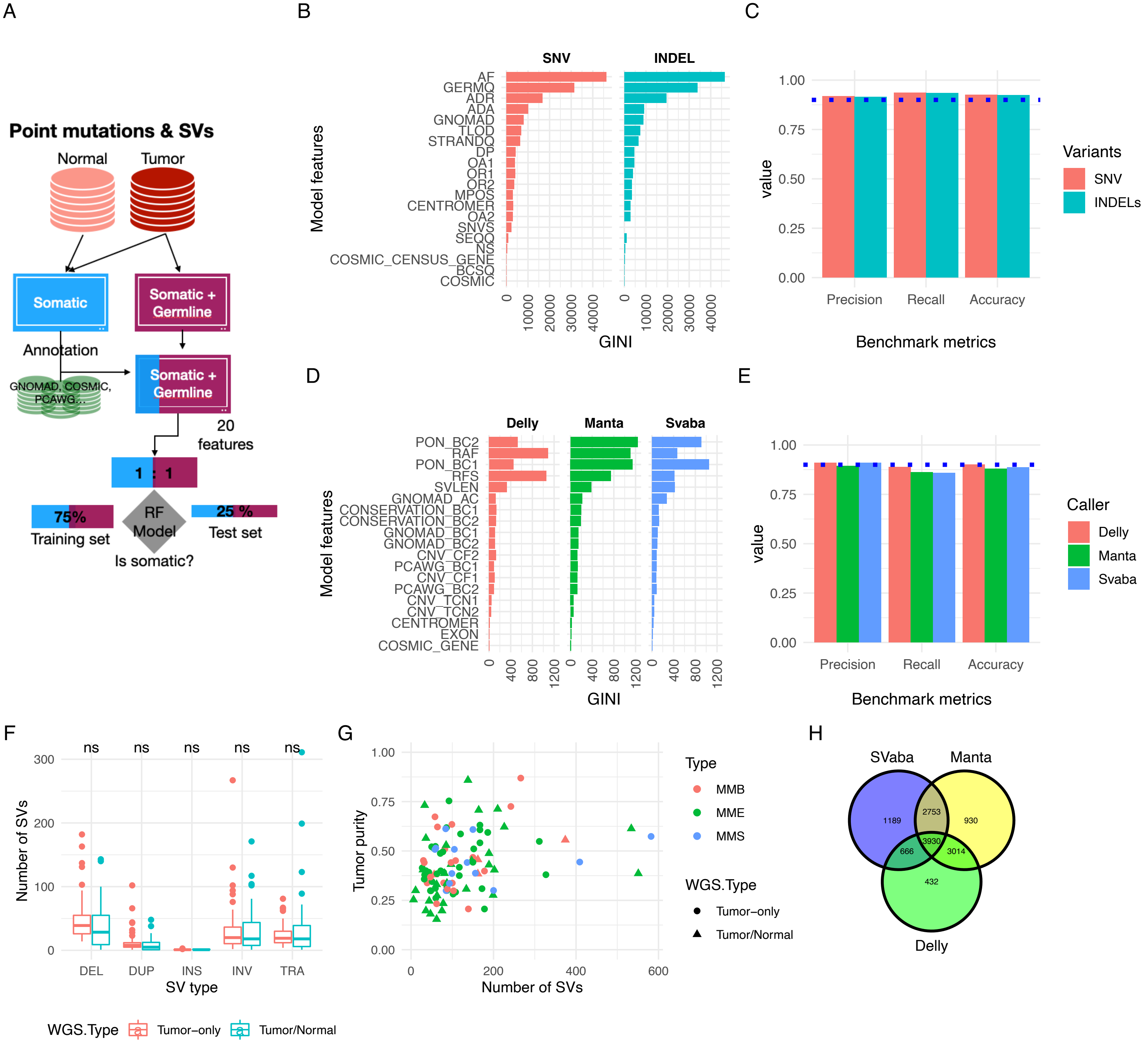

Figure 6

Click here to access/download;Figure;file-6.pdf

Figure 6

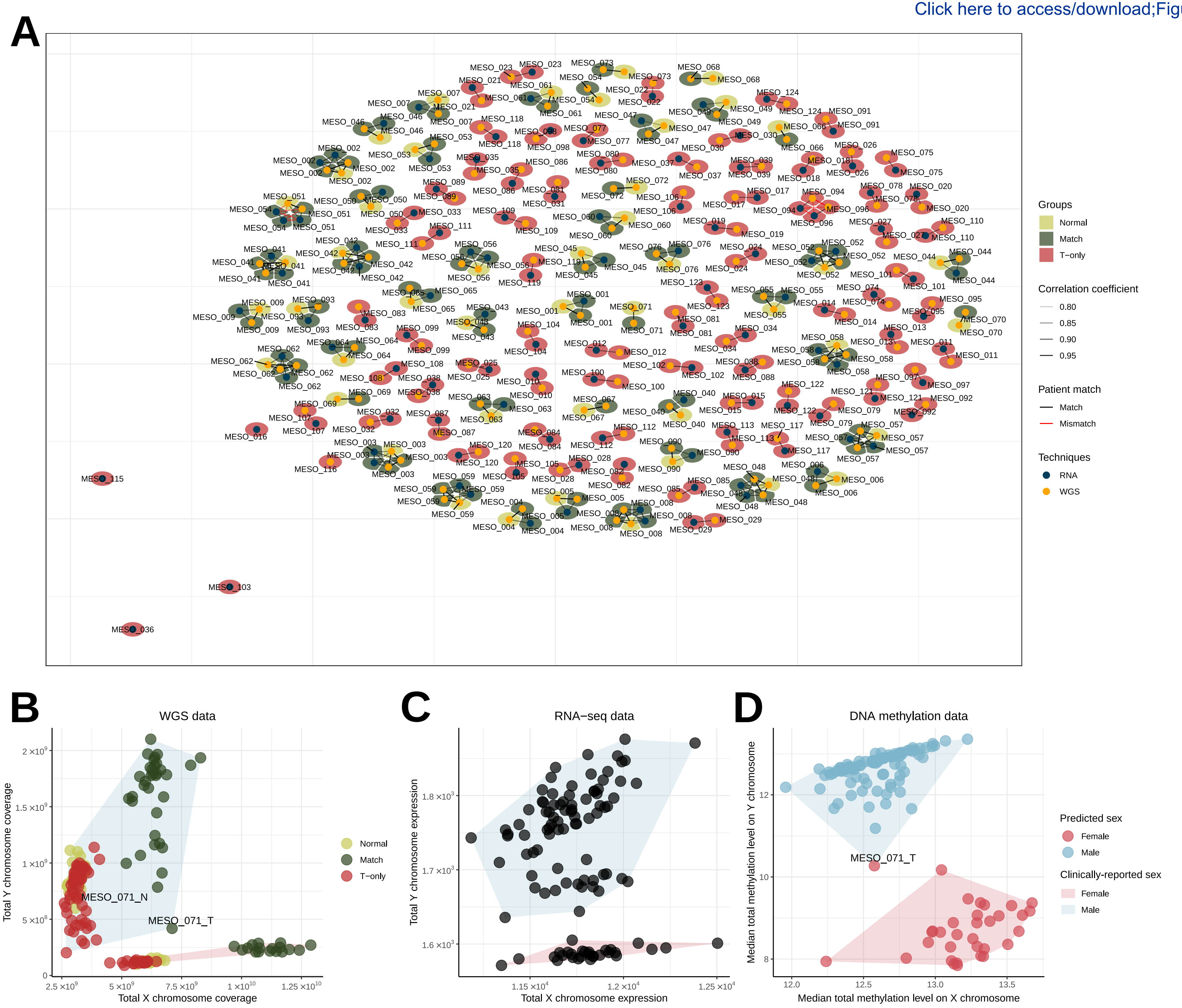

Figure 7

Click here to access/download;Figure;file-7.pdf

Figure 7

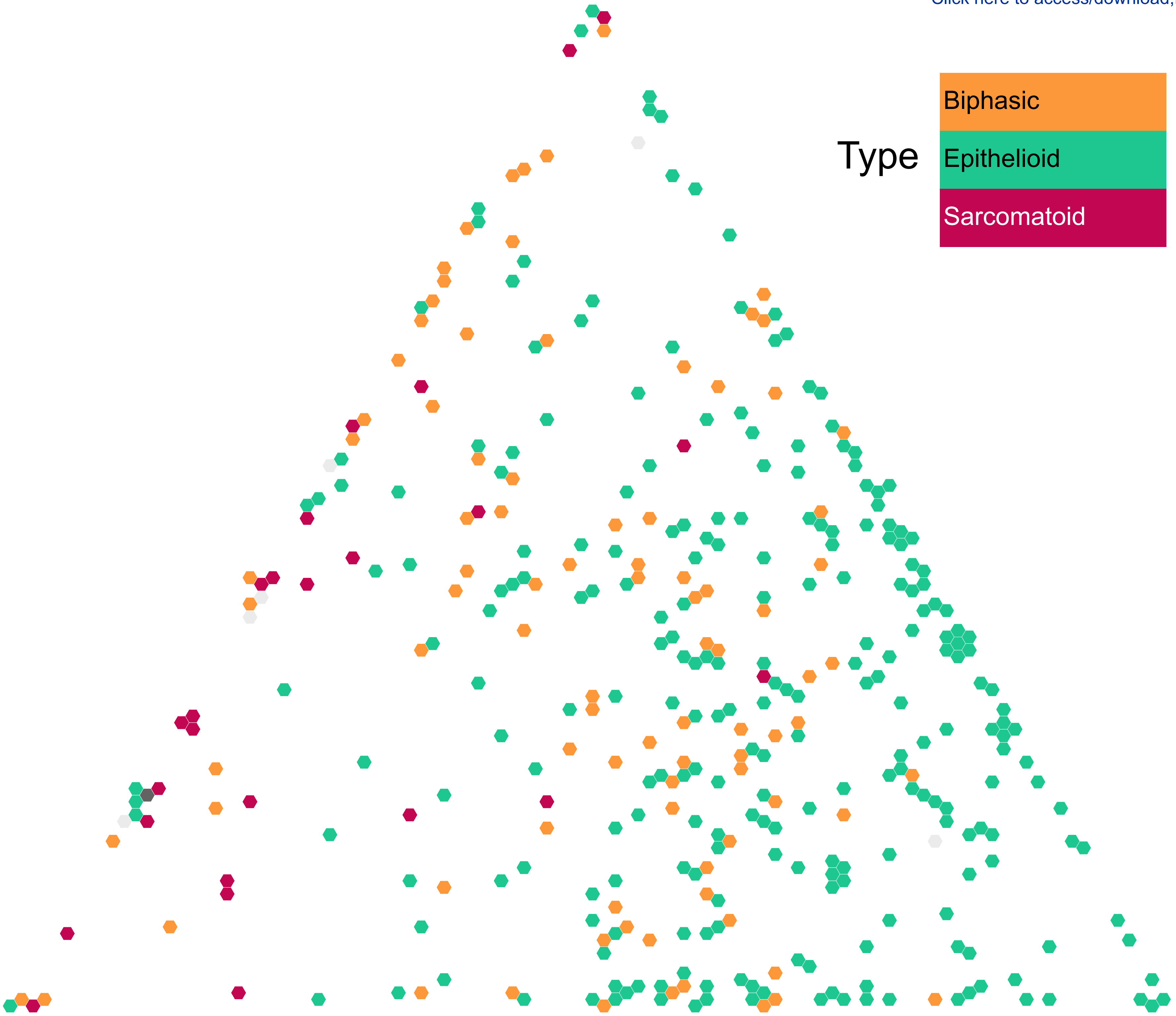

Figure S1

Click here to access/download
Supplementary Material
supp-1.pdf

Table S1

Click here to access/download
Supplementary Material
supp-2.tsv

Table S2

Click here to access/download
Supplementary Material
TableS2.xlsx

Supplementary Note 1

Click here to access/download
Supplementary Material
Supplementary_Note1.docx
