# Supplementary material for: A molecular phenotypic map of malignant pleural mesothelioma
Source: Gigascience. 2023 Jan 27;12:giac128. doi: 10.1093/gigascience/giac128 (PMC9881451; doi:10.1093/gigascience/giac128)
Supplement: giac128_Supplemental_Files [file giac128_supplemental_files.zip › Supplementary_Note1.docx]

**Supplementary Note 1: “Point Mutation and structural variant calling of tumor-only MPM samples”**

***Point Mutations***

Unlike copy number variants whereby the software (PURPLE) directly generated highly accurate results in tumor-only mode without any post-processing, for point mutations direct outputs from the software (Mutect2, [[1]](https://paperpile.com/c/hpQQLb/YOnqL)) and typical filters (i.e. removing variants matching germline databases) did not remove at high accuracy the germline variants present in tumor-only WGS. Therefore, we trained and evaluated the performance of a supervised machine learning model based on a random forest (RF, [[2]](https://paperpile.com/c/hpQQLb/QbAKr)) for distinguishing germline from somatic variants in tumor-only WGS (**Figure 5A**).

The matched samples were used as input for training and evaluating the performance of the random forest (RF) model (**Figure 5A**). Point mutations were called using Mutect2 using our nextflow pipeline Mutect2-nf (https://github.com/IARCbioinfo/mutect-nf, v2.2b). To build the RF model, we chose a total of 20 features divided into three main classes, namely: associated with external databases, genomic location/impact, and features obtained directly from the Mutect2 variant caller (**Figure 5B**). For the first class of features, we used the gnomAD (r3.0 with 526M SNPs and 69M indels) [[3]](https://paperpile.com/c/hpQQLb/HSUBW) and COSMIC (v90, with 18.6M SNVs and 0.99M indels) [[4]](https://paperpile.com/c/hpQQLb/RVkJx) databases as reference for germline and somatic variants, respectively. The GNOMAD feature encodes the minor allelic frequency of each variant matching a variant present in the gnomAD database, otherwise providing a value of 0. The matching of variants was performed using bcftools (v1.10.2, annotate function) [[5]](https://paperpile.com/c/hpQQLb/pmnUn). The COSMIC and COSMIC_GENE_CENSUS features are Boolean variables encoding if the variant matched one present in the COSMIC database or if its genomic location overlaps with the boundaries of a COSMIC gene (*n*=723). The second class of features included substitution patterns, functional impact, and genomic location of variants. The substitution patterns (i.e. mutation C->A) were converted into six categorical values (SNVS model feature), as somatic variants are often enriched in particular signatures [[6]](https://paperpile.com/c/hpQQLb/IEKd0). The BCSQ feature encodes the impact of variants into four categories (MODIFIER, LOW, MODERATE, and HIGH) according to how the mutation affects coding genes. Variant impact was calculated using the bcq [[7]](https://paperpile.com/c/hpQQLb/HnQdQ) function of bcftools and the classification of impact was performed with the Ensembl variant consequences table (https://www.ensembl.org/info/genome/variation/prediction/predicted_data.html). The CENTROMERE feature indicates if a variant is located in centromeric regions. Finally, fourteen features were derived from the Mutect2 caller, which are associated with read depth and orientation (DP, ADR, ADA, OR1, OR2, OA1, and OA2), sequencing errors and artifacts (MPOS, STRANDQ, SEQQ, TLOD, GERMQ), frequency across samples (NS), and allele frequency (AF, **Figure 5B**).

For training the RF model a total of 46 tumors with matched normal MPM whole-genome sequences called with both the tumor-only and matched modes of Mutect2 were used (**Figure 5A**). The matched somatic calls (ground-truth) were used to annotate the variants of the tumor-only WGS into germline and somatic classes. The number of germline variants called in tumor-only mode (*n*=694,273) greatly exceeded the number of somatic variants (*n*=203,993) generating an imbalance between classes. Therefore, we subsampled the germline class as a function of the somatic class to mitigate bias arising from class imbalance during training (1:1 somatic:germline ratio, *n*=407,984). To determine optimal values for the RF parameters we performed a grid search for tuning the mtry (4, 8, 12, 16), ntree (500, 1000, 1500), and nodesize (5, 25, 50, 100) parameters in a total of 48 RF-models. The training and evaluation of models was performed using 75% and the remaining 25% of the dataset, respectively. The grid search revealed that the optimum parameters were mtry=8, ntree=1000, and nodesize=5, reaching a model accuracy of 0.9276 in the testing set. Additionally, the minimum accuracy reached by any RF model was 0.9220, indicating that the parameter optimization had a marginal impact on the RF models. A random forest model for SNVs (rfvs01) was trained with the optimum parameters using a total of 326,388 (80%) variants (1:1 ratio). Analysis of the feature importance revealed that the allele frequency (AF) is the most discriminative feature included in the model (**Figure 5B**). For indels, a random forest model (rfvi01) was built with the same optimal parameters using a total of 337,442 variants (1:1 ratio, including 305,988 SNVs and 31,454 indels) and removing the SNVs feature. The performance of the optimal RF-models for SNVs and indels reached an accuracy of 0.926 and 0.924, respectively (**Figure 5C**). To further control the false positive rate (overall FDR=6.4%) we used different cut-offs (RF probability) to classify as somatic coding (>0.5) and non-coding (>0.75) variants. Finally, the trained RF models (rfvs01 and rfvi01) were used to classify a total of 1,454,942 variants (SNVs=1,317,200 and indels=137,742) of which 217,436 variants (including SNVs and indels) were classified as somatic. With these results we have developed a highly accurate and robust methodology to call SNVs and indels in tumor-only WGS datasets for which a series of matched tumor-normal samples are available. The source code and the random forest models implemented are available in the Github repository at https://github.com/IARCbioinfo/RF-mut-f.

***Structural Variants***

Large genomic rearrangements were detected using a consensus variant calling approach including SvABA (v1.1.0)[[8]](https://paperpile.com/c/hpQQLb/YWGa1), Manta (v1.6.0)[[9]](https://paperpile.com/c/hpQQLb/n1XFL), and Delly (v0.8.3)[[10]](https://paperpile.com/c/hpQQLb/uR6zf) followed by subsequent integration with SURVIVOR (v1.0.7)[[11]](https://paperpile.com/c/hpQQLb/SPgRD). Our nextflow pipeline implementing the consensus variant calling approach for matched WGS is available at https://github.com/IARCbioinfo/sv_somatic_cns. In brief, each structural variant caller was run following the recommended practices (filters and excluding masked genomic regions). Consensus between callers was built using SURVIVOR (merge command) by computing an all-versus-all structural variants (SVs) comparison. Matching pass-filter somatic SV pairs were considered when both breakpoints overlap at a maximum distance of 1kb. Somatic SVs called by at least two callers as well as single-tool SVs supported by > 15 pair-end reads were included in the consensus set.

Like for point mutations, we implemented custom random forest models to distinguish at high accuracy somatic from germline SVs in tumor-only MESOMICS samples (**Figure 5A**). The RF-models were composed of a total of 19 features based on external databases, a custom panel of normal, genomic regions, and SV features obtained directly from each SV caller (**Figure 5D**). The gnomAD database (v2.1, *n*=299,211) GRCh38 liftover (dbVar https://ftp.ncbi.nlm.nih.gov/pub/dbVar/data/Homo_sapiens/by_study/vcf/nstd166.GRCh38.variant_call.vcf.gz) was used as reference for germline SVs. Features describing the frequency of the SV in gnomAD (GNOMAD_AC) and the number of germline SVs around each breakpoint (10kb window, GNOMAD_BC1 and GNOMAD_BC2) were included in the RF SV model. The PCAWG consensus call set for SVs (v1.6, *n*=309,246) [[12]](https://paperpile.com/c/hpQQLb/k7qrh) was used as a reference of somatic SVs. The PCAWG SVs are in hg19 genome coordinates, thus we performed a liftover to GRCh38 using CrossMap (v0.3.9) [[13]](https://paperpile.com/c/hpQQLb/NOtSV). PCAWG SVs at sample level (*n*=2,748) were merged into a non-redundant cohort callset with SURVIVOR (merge subcommand) leading to a total of 283,980 non-redundant somatic SVs. Two features associated with the number of somatic SVs around each breakpoint (10kb window, PCAWG_BC1 and PCAWG_BC2) were included in the model. To further enhance the germline filtering we generated a custom panel-of-normal (PON) for each SV caller using our set of normal samples (*n*=46). A total of 56,572, 52,915, and 24,381 non-redundant PON SVs were collected for Manta, Delly and SvABA, respectively. Two features associated with the number of PON SVs around each breakpoint (10kb window, PON_BC1 and PON_BC2) were included in the model. For genomic regions, we annotated the SV breakpoints with features associated to known cancer genes (COSMIC_GENE, cosmic v90), coding exons (EXON, gencode v33), centromeres (CENTROMER), and conserved genomic regions (100-way PhastCons) [[14]](https://paperpile.com/c/hpQQLb/V3CHw). Finally, we included in the model features indicating the total copy number state (CNV_TCN1 and CNV_TCN2 for both breakpoints), tumor cell fraction estimations (CNV_CF1 and CNV_CF2 for both breakpoints), SV length (SVLEN), SV read depth (RFS), and SV alternative allele frequency (RAF). Our hypothesis behind all the aforementioned features is that the underlying genomic context of somatic SVs is different from those of germline SVs and that a random forest model should be able to distinguish both SV classes using the proposed features.

The training (75%) and evaluation (25%) of the random forest model for each SV caller was performed using a total of 12,454, 16,720, and 12,264 SVs at 1:1 somatic:germline proportions for Delly, Manta, and SvABA, respectively. All three SV random forest models were trained using the default random forest parameters (mtry=4, ntree=400 and nodesize=1). The precision, recall, and accuracy achieved by each model were 0.905±0.009, 0.87±0.016, and 0.889±0.010, respectively (**Figure 5E**). The most important features of the models were the number of PON SVs around both breakpoints, SV alternative allele frequency, SV read depth, and SV length (**Figure 5D**). To control the FDR, SVs located in coding and non-coding genomic regions were classified as somatic if their random-forest probability exceeded 0.5 and 0.75, respectively. Additionally, somatic SVs matching SVs present in the MESOMICS PON or located in centromeric regions were discarded. A consensus of SV sets for each tumor-only sample was built using the same steps performed for the matched WGS. Finally, SVs having a frequency higher than four across all the tumor-only samples were also classified as potentially germline and removed from the final consensus set. We performed additional comparisons by SV type, SV length, and number of SVs as a function of the purity of samples, WGS type(Tumor/Normal, Tumor-only) and MPM subtype and did not observe any significant difference between SVs called in the tumor-only or matched WGS MESOMICS series (**Figure 5F** and **5G**).

The SV calls for the MESOMICS tumor-only samples include a total of *n*=8,229 SVs, which combined with the SVs called in the matched series gave a total of *n*=12,914 (**Figure 5H**). With these results we have developed a highly accurate and robust methodology to call SVs in tumor-only WGS datasets for which a series of matched tumor-normal samples are available. The source code and the SV random forest models are available in the Github repository at https://github.com/IARCbioinfo/ssvht.

**References**

1. Benjamin D, Sato T, Cibulskis K, Getz G, Stewart C, Lichtenstein L. Calling Somatic SNVs and Indels with Mutect2. bioRxiv.

2. Liaw A, Wiener M, Others. Classification and regression by randomForest. *R news*. 2:18–222002;

3. Karczewski KJ, Francioli LC, Tiao G, Cummings BB, Alföldi J, Wang Q, et al.. The mutational constraint spectrum quantified from variation in 141,456 humans. *Nature*. 581:434–432020;

4. Tate JG, Bamford S, Jubb HC, Sondka Z, Beare DM, Bindal N, et al.. COSMIC: the Catalogue Of Somatic Mutations In Cancer. *Nucleic Acids Res*. 47:D941–72019;

5. Danecek P, Bonfield JK, Liddle J, Marshall J, Ohan V, Pollard MO, et al.. Twelve years of SAMtools and BCFtools. *Gigascience*. 2021; doi: 10.1093/gigascience/giab008.

6. Alexandrov LB, Kim J, Haradhvala NJ, Huang MN, Tian Ng AW, Wu Y, et al.. The repertoire of mutational signatures in human cancer. *Nature*. 578:94–1012020;

7. Danecek P, McCarthy SA. BCFtools/csq: haplotype-aware variant consequences. *Bioinformatics*. 33:2037–92017;

8. Wala JA, Bandopadhayay P, Greenwald NF, O’Rourke R, Sharpe T, Stewart C, et al.. SvABA: genome-wide detection of structural variants and indels by local assembly. *Genome Res*. 28:581–912018;

9. Chen X, Schulz-Trieglaff O, Shaw R, Barnes B, Schlesinger F, Källberg M, et al.. Manta: rapid detection of structural variants and indels for germline and cancer sequencing applications. *Bioinformatics*. 32:1220–22016;

10. Rausch T, Zichner T, Schlattl A, Stütz AM, Benes V, Korbel JO. DELLY: structural variant discovery by integrated paired-end and split-read analysis. *Bioinformatics*. 28:i333–92012;

11. Jeffares DC, Jolly C, Hoti M, Speed D, Shaw L, Rallis C, et al.. Transient structural variations have strong effects on quantitative traits and reproductive isolation in fission yeast. *Nat Commun*. 8:140612017;

12. Li Y, Roberts ND, Wala JA, Shapira O, Schumacher SE, Kumar K, et al.. Patterns of somatic structural variation in human cancer genomes. *Nature*. 578:112–212020;

13. Zhao H, Sun Z, Wang J, Huang H, Kocher J-P, Wang L. CrossMap: a versatile tool for coordinate conversion between genome assemblies. *Bioinformatics*. 30:1006–72014;

14. Pollard KS, Hubisz MJ, Rosenbloom KR, Siepel A. Detection of nonneutral substitution rates on mammalian phylogenies. *Genome Res*. 20:110–212010;
