# Supplementary figures and images for: A molecular phenotypic map of malignant pleural mesothelioma
Source: Gigascience. 2023 Jan 27;12:giac128. doi: 10.1093/gigascience/giac128 (PMC9881451; doi:10.1093/gigascience/giac128)

Figure S1

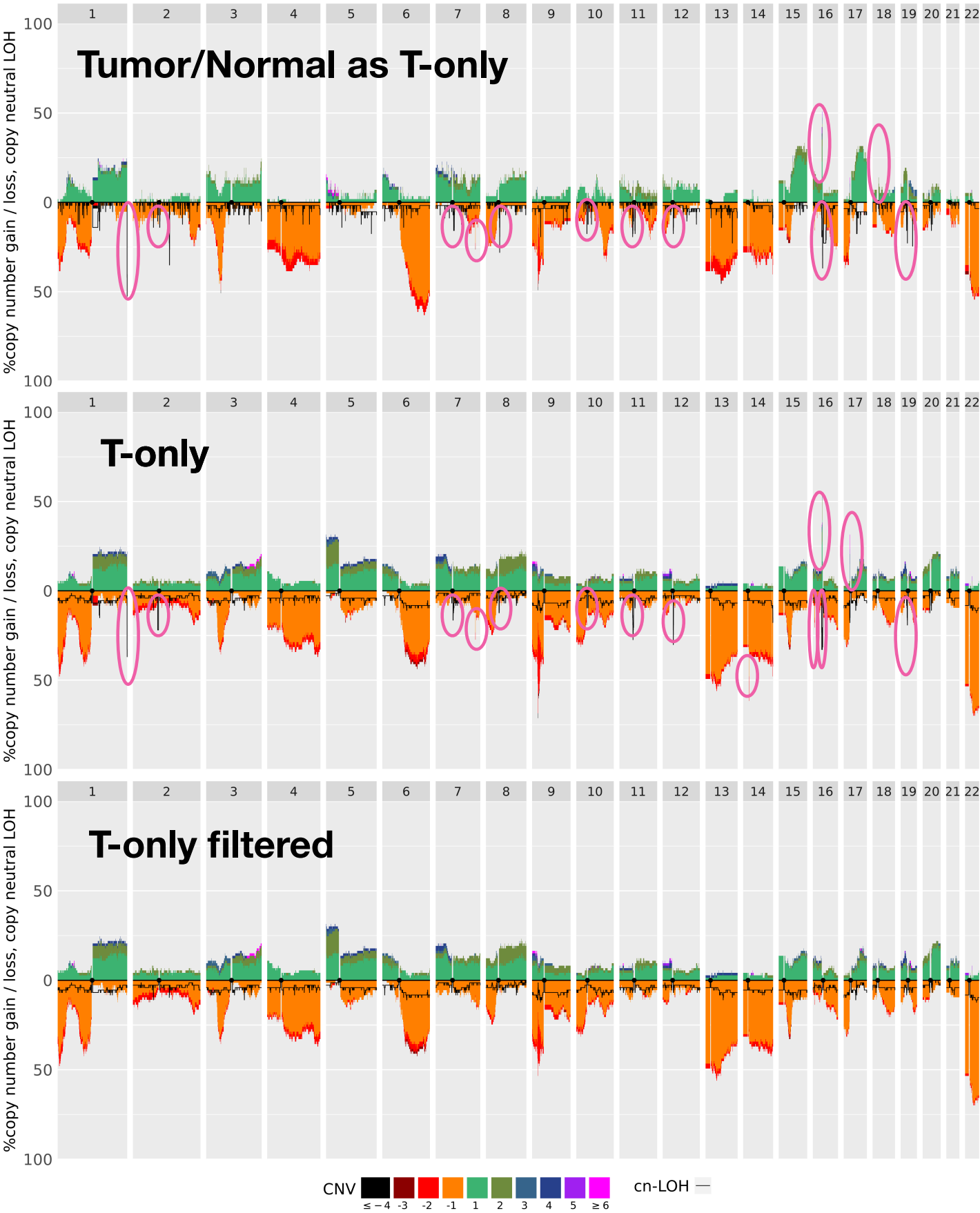

Supplement: giac128_Supplemental_Files [file giac128_supplemental_files.zip › supp-1.pdf]
