# Supplementary material for: A molecular phenotypic map of malignant pleural mesothelioma
Source: Gigascience. 2023 Jan 27;12:giac128. doi: 10.1093/gigascience/giac128 (PMC9881451; doi:10.1093/gigascience/giac128)
Supplement: giac128_Response_to_Reviewer_Comments_Original_Submission [file giac128_response_to_reviewer_comments_original_submission.pdf]

Author's Response To Reviewer Comments

Close

Dear Dr Nogoy,

We would like to thank you for the prompt and thorough review of our article entitled "A molecular phenotypic map of Malignant Pleural Mesothelioma", and for giving us the possibility of addressing the reviewers' comments. We have now generated a revised version of the manuscript taking into account all suggestions. We have also addressed the editor's and reviewers' comments point-by-point, below. We have highlighted changes in the main text in yellow for easier reference.

Please note that this Data Note is aimed to be published in coordination with or after the publication of our analysis paper, which has just been accepted in Nature Genetics. We apologize it took us more time than we expected to revise the manuscript because we had to work on both papers in parallel.

We very much look forward to your consideration of our revised manuscript for publication in GigaScience.

Best regards,

Matthieu

Reviewer #1:

Authors did a fantastic job by integrating MPM multi-omics datasets and created an integrative and interactive map for users to explore these datasets. MPM is a rare cancer type and understudied so such resources are very useful to move the field forward at a molecular level. The comprehensive data is well presented and the manuscript is well written to explain the complex genomics dataset for MPM.
All the figures are well explained and very clear to understand

Answer: We thank the reviewer for his enthusiasm, the useful comments, for noting the value of our work and how it will contribute to move forward MPM research.

Minor point:
- Author mentioned an evaluation of tumor purity was done using pathological review, did author used molecular data such as genomic data to find tumor purity ? and if yes, how was the consensus ? This is very important factor to interpret the genomic results as the data was sequenced at 30X
- In the same line, RNAseq can also be used to identify tumor purity and it will be really helpful for users to clear picture on tumor purity.

Answer: We now report purity estimates from genomic and expression data in Table S2, and show their correlation with the pathological review estimates in a new "Purity" subsection of "Data Validation" p. 11.

- Is it not very clear from method section that the same MPM samples were used to sequence at DNA , RNA and DNA methylation level ? A brief explanation or table will be very easy for users to understand.

Answer: We now mention in the text (beginning of Data Description section p. 3) that a vast majority of the samples (105/120) had complete data (WGS, RNA-seq and methylation), and provide in Table S2 the list of samples with each omic data available.

- Recent WHO classify MPM into three different histopathological types. Did author do any unsupervised analysis from these comprehensive data to understand MPM heterogeneity or replicate WHO classification? or did author find WHO subtypes of MPM using molecular dataset ? A brief analysis/comment on usage of

histological classification Vs Molecular classification will certainly move the MPM research field forward as researcher have found vast differences between histological vs molecular classification and the field is moving towards more molecular based classification in clinic.

Answer: This paper is a Data Note to be published in coordination with or after the publication of our analysis paper, in which we indeed performed these in depth analyses (Mangiante et al. 2021, preprint available at https://www.biorxiv.org/content/10.1101/2021.09.27.461908v1, ref 10 in the manuscript, now accepted for publication in Nature Genetics). We apologize this was unclear, and we have now added a sentence at the beginning of the Data Description (p. 4) section referring to this paper.

Reviewer #2:

In this paper, the authors describe a new public resource for the molecular characterization of malignant pleural mesothelioma (MPM), which they describe as the most comprehensive to date. They perform WGS, transcriptome, and methylation arrays for 120 patients with MPM sourced through the MESOMICS project and integrate this dataset with an additional several hundred patients from previously published datasets.

Although I cannot independently verify their claim that this is the largest and most comprehensive dataset for MPM, it is quite impressive and expansive. The pipeline utilized is well described and the results at all stages are transparently shared for prospective users of this dataset.

Answer: We thank the reviewer for his/her review, and in particular for noting the importance of transparent sharing of the data processing and analysis.

The description of the methods to identify and remove germline variants is interesting, although the length somewhat detracts from the main goal of the paper in describing an MPM resource. Perhaps, this part could be condensed with the technical details presented in supplement. This comment pertains to both the Point Mutations and Structural Variants sections.

Answer: We have followed the reviewer suggestion and have condensed (from 1610 to 757 words) this part of the methods and put the more technical details in the supplementary Note 1.

Additional moderate concerns:

There are insufficient details provided on the clinical and epidemiological parameters. Indirectly, it would appear that sex, age class, and smoking status are the clinical parameters - but what are the age classes? Is smoking status binary ever/never, or more involved? There ought to be a data dictionary provided as a supplemental table which describes each clinical/epidemiological variable, along with the possible values that the variable can take on. It should additionally be explained why other important clinical parameters are not available - most importantly, the presence of accompanying pulmonary comorbidity such as chronic obstructive pulmonary disease (COPD) and the existence of conditions that might preclude the use of standard systemic therapies, such as renal disease precluding the use of platinum agents.

Answer: We apologize for the lack of clarity surrounding the clinical data and now provide more details about the clinical and epidemiological characteristics of the cohort. We have added a data dictionary for Table S2 (2nd worksheet tab in the Excel file), and now mention it on p. 4. We also explicitly state which clinical data is available on p. 4, in particular mentioning that we “provide basic clinical data (age, sex, survival) as well as exposure (asbestos, smoking), and treatment data (usage and type of chemotherapy, surgery, radiotherapy, and precision treatment),” and that “Comorbidity data were not available, however we provide symptoms that are informative on the state of the patient at diagnosis (pain, pleural effusion, dyspnea, pneumothorax, coughing).”

Context: I would like to see more here about the role of asbestos in the etiology, including what might be known about the pathophysiology of asbestos fibers at the molecular level. Also, there is nothing here about the evolution of treatment for MPM; the latest "state-of-the-art" regimens (platinum doublet + bevacizumab [MAPS; NCT00651456] and dual checkpoint inhibition [Checkmate 743; NCT02899299]) have reported median survival in the 18-month range, which is distinctly better than the median survivals

quoted by the authors. Finally, I would like to see one or more direct references to the clinical trials where molecular heterogeneity has "fueled the implementation of drug trials for more tailored MPM treatments".

Answer: This paper is a Data Note to be published in coordination with or after the publication of our analysis paper, in which we discuss the effect of asbestos fiber at the molecular level (Mangiante et al. 2021, preprint available at https://www.biorxiv.org/content/10.1101/2021.09.27.461908v1, ref 10 in the manuscript, now accepted for publication in Nature Genetics). We apologize this was unclear, and we have now added a sentence at the beginning of the Data Description (p. 4) section referring to this paper.

We now mention p. 4 that a vast majority of patients were treated with chemotherapy, surgery, or radiotherapy and a single patient received immunotherapy (now in Table S2). We also mention that because of the retrospective nature of the samples from the French MESOBANK, patients were diagnosed (year of diagnosis [1998-2017], median of 2011) and treated (year of death or end of follow-up [2000-2020], median of 2013) before the results from the MAPS (2016) and Checkmate 743 trials (2021), and before the authorization of nivolumab and ipilimumab by the European Medicines Agency in 2022 (note that despite the MAPS trial, bevacizumab is not a standard first line treatment in France); we thus indicate to the readers that future studies will hopefully report longer survivals as the reviewer mentions, and cite these clinical trials.

Data Description: All specimens in the MESOMICS study are said to be collected from surgically resected MPM; this also appears to be the case for the integrated multi-omic studies from Bueno et al. and Hmeljak et al. and this should be explicitly indicated. Somewhere, it should also be explicitly discussed that this is an important limitation in the future utility of this data - surgical specimens are convenience samples and while they do provide important information, they lack treatment exposure. Given that many if not most patients with MPM will survive to 2nd or 3rd line systemic therapy, and that 1st line is fairly standardized, a knowledge of induced mutations is going to be essential to the ultimate goal of precision medicine.

Answer: We thank the reviewer for this important comment. All specimens in the MESOMICS study are collected from surgically resected MPM, this is also the case for the majority of samples from the Bueno et al. and Hmeljak et al studies. Specifically, in Hmeljak et al. frozen primary tumors were obtained from surgical resection (n=55), biopsy (n=9), or unknown surgery type (n=10), and in Bueno et al. fresh-frozen samples were obtained from patients undergoing extirpative surgery for malignant pleural mesothelioma. This information has now been reported on p. 12 and added to Table S2. We have also added the important point that future studies will be needed to describe the molecular landscape of MPM after 1st and 2nd line of systemic therapy to develop effective precision medicine strategies (final conclusion sentence of the manuscript p. 14).

Minor concerns:

The labels in the figures (e.g., Figure 2 - "Unmapped..too.short") are human-readable but could still be presented in a more friendly fashion. All acronyms should be defined.

Answer: We have updated the labels by removing the dots and underscores, thanks for spotting this error.

Reviewer #3:

I am reviewing only process of obtaining access to the controlled data described in your study.
You have been very prompt and clear in all communication regarding the DAA and access to EGAS00001004812 was promptly granted by the EGA.
As you are aware I am having difficulty actually downloading the 3 datasets, but I am confident the issues will be resolved soon.

Answer: We thank you for carefully going through the entire data access process to ensure that the data is available. We now provide more information about the process of requesting access and setting up the download of the data p. 14.

Close
