# Supplementary material for: A molecular phenotypic map of malignant pleural mesothelioma
Source: Gigascience. 2023 Jan 27;12:giac128. doi: 10.1093/gigascience/giac128 (PMC9881451; doi:10.1093/gigascience/giac128)
Supplement: giac128_Response_to_Reviewer_Comments_Revision_1 [file giac128_response_to_reviewer_comments_revision_1.pdf]

Author's Response To Reviewer Comments

Close

Dear Dr. Nogoy,

Please find attached the updated version of our manuscript following your requests:
- All URLs from the main text are now references
- I completed the Availability of source code section
- I added some missing RRIDs
- I fixed a typo in the URL in the abstract
- Ref 10 is now "Nature Genetics in press" instead of a preprint.

Best wishes,

Matthieu Foll

Close
