# Supplementary material for: A molecular phenotypic map of malignant pleural mesothelioma
Source: Gigascience. 2023 Jan 27;12:giac128. doi: 10.1093/gigascience/giac128 (PMC9881451; doi:10.1093/gigascience/giac128)
Supplement: giac128_Reviewer_1_Report_Original_Submission — Saurabh V Laddha -- 8/23/2022 Reviewed [file giac128_reviewer_1_report_original_submission.pdf]

Reviewer Report

Title: A molecular phenotypic map of Malignant Pleural Mesothelioma

Version: Original Submission **Date:** 8/23/2022

Reviewer name: Saurabh V Laddha

Reviewer Comments to Author:

Authors did a fantastic job by integrating MPM multi-omics datasets and created an integrative and interactive map for users to explore these datasets. MPM is a rare cancer type and understudied so such resources are very useful to move the field forward at a molecular level. The comprehensive data is well presented and the manuscript is well written to explain the complex genomics dataset for MPM.All the figures are well explained and very clear to understandMinor point:- Author mentioned an evaluation of tumor purity was done using pathological review, did author used molecular data such as genomic data to find tumor purity ? and if yes, how was the consensus ? This is very important factor to interpret the genomic results as the data was sequenced at 30X- In the same line, RNAseq can also be used to identify tumor purity and it will be really helpful for users to clear picture on tumor purity.- Is it not very clear from method section that the same MPM samples were used to sequence at DNA , RNA and DNA methylation level ? A brief explanation or table will be very easy for users to understand.- Recent WHO classify MPM into three different histopathological types. Did author do any unsupervised analysis from these comprehensive data to understand MPM heterogeneity or replicate WHO classification? or did author find WHO subtypes of MPM using molecular dataset ? A brief analysis/comment on usage of histological classification Vs Molecular classification will certainly move the MPM research field forward as researcher have found vast differences between histological vs molecular classification and the field is moving towards more molecular based classification in clinic.

Level of Interest

Please indicate how interesting you found the manuscript: Choose an item.

Quality of Written English

Please indicate the quality of language in the manuscript: Choose an item.

Declaration of Competing Interests

Please complete a declaration of competing interests, considering the following questions:

- Have you in the past five years received reimbursements, fees, funding, or salary from an organisation that may in any way gain or lose financially from the publication of this manuscript, either now or in the future?

- Do you hold any stocks or shares in an organisation that may in any way gain or lose financially from the publication of this manuscript, either now or in the future?
- Do you hold or are you currently applying for any patents relating to the content of the manuscript?
- Have you received reimbursements, fees, funding, or salary from an organization that holds or has applied for patents relating to the content of the manuscript?
- Do you have any other financial competing interests?
- Do you have any non-financial competing interests in relation to this paper?

If you can answer no to all of the above, write 'I declare that I have no competing interests' below. If your reply is yes to any, please give details below.

I declare that I have no competing interests

I agree to the open peer review policy of the journal. I understand that my name will be included on my report to the authors and, if the manuscript is accepted for publication, my named report including any attachments I upload will be posted on the website along with the authors' responses. I agree for my report to be made available under an Open Access Creative Commons CC-BY license (http://creativecommons.org/licenses/by/4.0/). I understand that any comments which I do not wish to be included in my named report can be included as confidential comments to the editors, which will not be published.

Choose an item.

To further support our reviewers, we have joined with Publons, where you can gain additional credit to further highlight your hard work (see: https://publons.com/journal/530/gigascience). On publication of this paper, your review will be automatically added to Publons, you can then choose whether or not to claim your Publons credit. I understand this statement.

Yes Choose an item.
