# Supplementary material for: A molecular phenotypic map of malignant pleural mesothelioma
Source: Gigascience. 2023 Jan 27;12:giac128. doi: 10.1093/gigascience/giac128 (PMC9881451; doi:10.1093/gigascience/giac128)
Supplement: giac128_Reviewer_2_Report_Original_Submission — Jeremy Warner -- 8/31/2022 Reviewed [file giac128_reviewer_2_report_original_submission.pdf]

Reviewer Report

Title: A molecular phenotypic map of Malignant Pleural Mesothelioma

Version: Original Submission **Date:** 8/31/2022

Reviewer name: Jeremy Warner

Reviewer Comments to Author:

In this paper, the authors describe a new public resource for the molecular characterization of malignant pleural mesothelioma (MPM), which they describe as the most comprehensive to date. They perform WGS, transcriptome, and methylation arrays for 120 patients with MPM sourced through the MESOMICS project and integrate this dataset with an additional several hundred patients from previously published datasets.Although I cannot independently verify their claim that this is the largest and most comprehensive dataset for MPM, it is quite impressive and expansive. The pipeline utilized is well described and the results at all stages are transparently shared for prospective users of this dataset.The description of the methods to identify and remove germline variants is interesting, although the length somewhat detracts from the main goal of the paper in describing an MPM resource. Perhaps, this part could be condensed with the technical details presented in supplement. This comment pertains to both the Point Mutations and Structural Variants sections.Additional moderate concerns:There are insufficient details provided on the clinical and epidemiological parameters. Indirectly, it would appear that sex, age class, and smoking status are the clinical parameters - but what are the age classes? Is smoking status binary ever/never, or more involved? There ought to be a data dictionary provided as a supplemental table which describes each clinical/epidemiological variable, along with the possible values that the variable can take on. It should additionally be explained why other important clinical parameters are not available - most importantly, the presence of accompanying pulmonary comorbidity such as chronic obstructive pulmonary disease (COPD) and the existence of conditions that might preclude the use of standard systemic therapies, such as renal disease precluding the use of platinum agents.Context: I would like to see more here about the role of asbestos in the etiology, including what might be known about the pathophysiology of asbestos fibers at the molecular level. Also, there is nothing here about the evolution of treatment for MPM; the latest "state-of-the-art" regimens (platinum doublet + bevacizumab [MAPS; NCT00651456] and dual checkpoint inhibition [Checkmate 743; NCT02899299]) have reported median survival in the 18-month range, which is distinctly better than the median survivals quoted by the authors. Finally, I would like to see one or more direct references to the clinical trials where molecular heterogeneity has "fueled the implementation of drug trials for more tailored MPM treatments".Data Description: All specimens in the MESOMICS study are said to be collected from surgically resected MPM; this also appears to be the case for the integrated multi-omic studies from Bueno et al. and Hmeljak et al. and this should be explicitly indicated. Somewhere, it should also be explicitly discussed that this is an important limitation in the future utility of this data - surgical specimens are convenience samples and while they do provide important information, they lack treatment exposure. Given that many if not most patients with MPM will survive to 2nd or 3rd line systemic therapy, and that 1st line is fairly standardized, a knowledge of induced

mutations is going to be essential to the ultimate goal of precision medicine.Minor concerns:The labels in the figures (e.g., Figure 2 - "Unmapped..too.short") are human-readable but could still be presented in a more friendly fashion. All acronyms should be defined.

Level of Interest

Please indicate how interesting you found the manuscript: Choose an item.

Quality of Written English

Please indicate the quality of language in the manuscript: Choose an item.

Declaration of Competing Interests

Please complete a declaration of competing interests, considering the following questions:

- Have you in the past five years received reimbursements, fees, funding, or salary from an organisation that may in any way gain or lose financially from the publication of this manuscript, either now or in the future?
- Do you hold any stocks or shares in an organisation that may in any way gain or lose financially from the publication of this manuscript, either now or in the future?
- Do you hold or are you currently applying for any patents relating to the content of the manuscript?
- Have you received reimbursements, fees, funding, or salary from an organization that holds or has applied for patents relating to the content of the manuscript?
- Do you have any other financial competing interests?
- Do you have any non-financial competing interests in relation to this paper?

If you can answer no to all of the above, write 'I declare that I have no competing interests' below. If your reply is yes to any, please give details below.

I declare that I have no competing interests

I agree to the open peer review policy of the journal. I understand that my name will be included on my report to the authors and, if the manuscript is accepted for publication, my named report including any attachments I upload will be posted on the website along with the authors' responses. I agree for my report to be made available under an Open Access Creative Commons CC-BY license (http://creativecommons.org/licenses/by/4.0/). I understand that any comments which I do not wish to be included in my named report can be included as confidential comments to the editors, which will not be published.

Choose an item.

To further support our reviewers, we have joined with Publons, where you can gain additional credit to further highlight your hard work (see: https://publons.com/journal/530/gigascience). On publication of this paper, your review will be automatically added to Publons, you can then choose whether or not to claim your Publons credit. I understand this statement.

Yes Choose an item.
